# Supplementary material for: Astroglial dysfunction in models of CDKL5 deficiency disorder
Source: Front Cell Neurosci. 2026 Oct 30;20:1934251. doi: 10.3389/fncel.2026.1934251 (PMC13759366; doi:10.3389/fncel.2026.1934251)
Supplement: Supplementary file 1 [file Data_Sheet_1.pdf]

SUPPLEMENTARY INFORMATION

Supplemental Tables

TARGET	HOST	SOURCE	CLONALITY	DILUTION		
				WB	ICC	IHC
CDKL5	Mouse	Santa Cruz, Sc-376314	Monoclonal	1:500-1:1000	-	-
ALDH1L1	Rabbit	Abcam, ab190293	Polyclonal	-	1:500	-
AQP4	Rabbit	ProteinTech, 16473-AP	Polyclonal	1:1000 - 1:5000	1:100	-
AQP4	Rabbit	Alomone, 249-323	Polyclonal	-	1:2000	1:1000
GFAP	Rabbit	Abcam, ab68428	Polyclonal	1:1000 - 1:4000	1:250	-
GFAP	Chicken	Abcam, ab4674	Polyclonal	-	-	1:2000
GFP	Rabbit	Abcam, ab290	Polyclonal	-	-	1:1000
NeuN	Mouse	Abcam, ab104224	Monoclonal	-	-	1:1000
GAPDH	Rabbit	Cell Signalling, 2118	Monoclonal	1:2500	-	-
pEB2	Rabbit	Produced and provided by Dr. Sila Ultanir.	Polyclonal	1:500 - 1:2000	-	-
EB2	Rat	Abcam, ab45767	Monoclonal	1:500 – 1:1000	-	-
S100β	Rabbit	Abcam, Ab52642	Polyclonal	-	1:500	
CD44	Rabbit	Abcam, Ab243894	Monoclonal	-	1:500	-

Supplemental Table 1 – List of primary antibodies

NAME	SOURCE	DILUTION		
		WB	ICC	IHC
Donkey anti-Rabbit IgG H&L (HRP)	Abcam, ab16284	1:2000	-	-
Goat anti-Mouse IgG (H+L), Alexa Fluor™ 594	Invitrogen, A11032	-	1:1000	-
Goat anti-Rabbit IgG (H+L) Alexa Fluor™ 488	Invitrogen, A11034	-	1:1000	-
Peroxidase AffiniPure™ Goat anti-Mouse HRP	Jackson Laboratories, 115-035-003	1:10,000	-	-
Donkey anti-Rabbit IgG (H+L) Alexa Fluor™ 488	Invitrogen, A21206	-	-	1:500
Donkey anti-Mouse IgG (H+L) Alexa Fluor™ 568	Invitrogen, A10037	-	-	1:500
Alexa Fluor® 647 AffiniPure™ Donkey Anti-Chicken IgY (IgG) (H+L)	Jackson Laboratories, 703-605-155			1:500
Goat anti-Rat IgG (H+L), HRP	Invitrogen, 31470	1:5000	-	-

Supplemental Table 2 – List of Secondary Antibodies

Primary Author	DESCRIPTION	Number of Samples	Reference
Lorenzi	Cell line (Sciencell)	3	[60]
Voss	Isolated from foetal cortex	2	[61]
Zhang	Isolated from foetal cortex	5	[63]
Zhang	Isolated from adolescent temporal lobe	2	[63]
Zhang	Isolated from adult temporal lobe	2	[63]
Ma	Cell line (Sciencell)	6	[64]
Crowe	Cell line (Sciencell)	2	[65]
Li	Isolated from foetal cortex	2	[66]

Supplemental Table 3a – List of human bulk RNAseq datasets

Primary Author	Region	Age Category	Number of Samples	Reference
Lattke	Cortex	Juvenile	3	[68]
Lattke	Cortex	Young Adult	3	[68]
Erickson	Cortex	Young Adult	17	[69]
Cheng	Hippocampus	Juvenile	9	[67]
Cheng	Cortex	Juvenile	4	[67]
Roboon	Cortex	Juvenile	4	[70]
Schulte	Cortex	Juvenile	4	[71]
Schmid	Cortex	Juvenile	2	[72]

Supplemental Table 3b – list of mouse bulk RNAseq datasets

Primary Author	Region	Sequencing Method	Age	Species	Number of Cells	Reference
Yao	Isocortex and hippocampus	scRNAseq	P50-121	Mouse	1638333	[107]
Yao	Primary motor cortex	scRNAseq and snRNAseq	P52-59	Mouse	18905	[108]
Steuernagel	Hypothalamus	snRNAseq	Various	Mouse	59785	[75]
Batiuk	Hippocampus	scRNAseq	P53	Mouse	820	[76]
Batiuk	Cortex	scRNAseq	P53	Mouse	991	[76]

Hochgerner	Hippocampus	scRNAseq	P12-35	Mouse	5454	[77]
Siletti	Right Hemisphere	snRNAseq	29 – 60 years	Human	158449	[78]
Jorstad	Cortex	scRNAseq	24 - 57 years	Human	20759	[79]
Seeker	Primary motor cortex and cerebellum	snRNAseq	30 – 75 years	Human	768	[80]
Jakel	White matter	snRNAseq	35 – 82 years	Human	392	[81]
Phan	Caudate and Putamen	snRNAseq	23 – 60 years	Human	4195	[82]
Darmanis	Cortex (anterior temporal lobe)	scRNAseq	21 – 63 years	Human	466	[83]

Supplemental Table 3c – List of sc/snRNAseq datsets

NAME	5' TO 3' SEQUENCE
CDKL5 shRNA	GATCCCCGGG ACAAAGTACC TCAAGTCAAA TTCAAGAGAT TTGACTTGAG GTACTTTGTC CCGGTTTTTT ACGCGTG
Scrambled shRNA	GATCCGAACA ATCCCAGGAG ACGCACTAAT TTCAAGAGAA TTAGTGCGTC TCCTGGGATT GTTCTTTTTT ACGCGTG

Supplemental Table 4 – 5' to 3' shRNA construct cloned into AAV transfer plasmid. Green = sticky ends; orange = siRNA target sequence and reverse complement; red = loop sequence; blue = termination signal.

Case Number	Age (pcw)	Brain Region	Experiment
FT20	17	Occipital Ctx.	Immunostaining
FT21	17	Occipital Ctx.	Immunostaining
FT24	17	Occipital Ctx.	Immunostaining
FT32	17	Occipital Ctx.	Transduced with shRNA/Scr AAV, MEA, protein and RNA analysis
FT27	18	Occipital Ctx.	Transduced with shRNA/Scr AAV, protein and RNA analysis
FT36	18	Frontal Ctx	Transduced with shRNA/Scr AAV, protein and RNA analysis
FT37	16	Occipital Ctx	Transduced with shRNA/Scr AAV, RNA analysis

Supplemental Table 5 – Age and region of foetal brain tissue utilised in the current study.

Case Number	Age (yrs)	Brain Region	Sex	Experiment
CE94	71	Right temporal cortex	Male	Transduced with shRNA/Scr AAV. Immunostaining, protein analysis.
CE95	60	Left temporal cortex	Female	Transduced with shRNA/Scr AAV, MEA, protein and RNA analysis
CE97	56	Right temporal cortex	Male	Transduced with shRNA/Scr AAV, MEA, protein and RNA analysis
CE98	30	Inferior temporal gyrus	Female	Transduced with shRNA/Scr AAV, Protein analysis

Supplemental Table 6 – Age and region of adult cortical brain tissue utilised in the current study.

Supplemental Figures

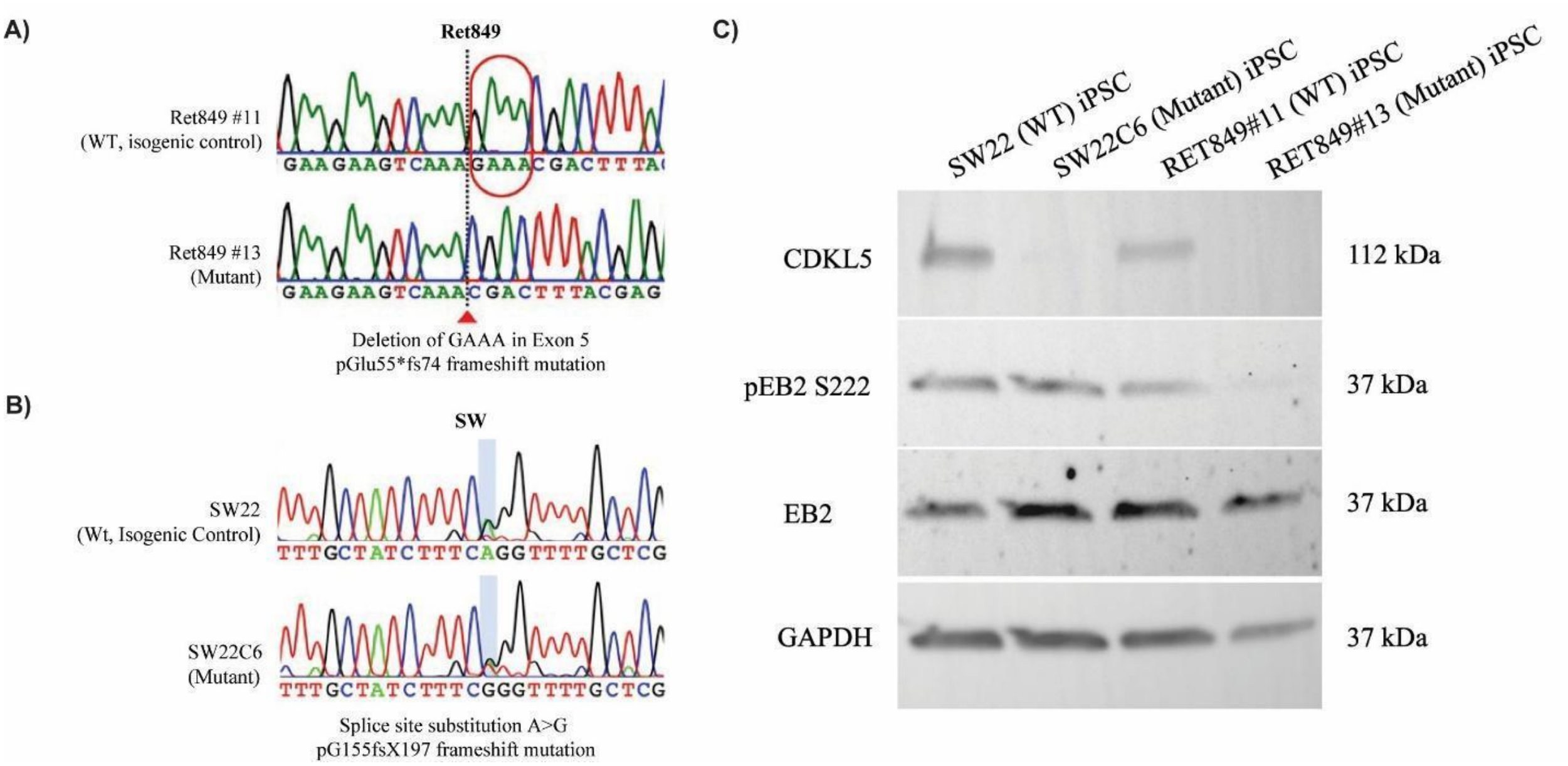

Supplemental Figure 1: CDKL5 gene variants in iPSCs

A) Sequencing of RT-PCR products generated using RNA extracted from Ret849 #11 and #13 iPSCs and located in exon 5 of *CDKL5*. Ret849 #13 has a deletion of GAAA (represented by red circle in Ret849 #11), resulting in a pGlu55*fs74 frameshift mutation. Also appears in Gao et al. 2020 [28]. **B)** Sequencing of PCR product of genomic *CDKL5*, generated from DNA extracted from SW22 and SW22C6 iPSCs. SW22C6 has a deletion at the splice acceptor site for exon 8, highlighted by red line and resulting in a pG155fsX197 frameshift mutation. The A>G substitution at position 298 (B highlighted) in the mutant amplicon was confirmed using different base calling software at AZENTA Life Sciences. **C)** Diminished CDKL5 protein expression in SW22C6 and Ret849 #13 iPSC cell lines was confirmed via Western Blot. Protein from WT and CDKL5 mutant iPSCs was probed for CDKL5, phosphorylated EB2 (pEB2) at Serine 222 (S222), EB2 and GAPDH as a loading control.

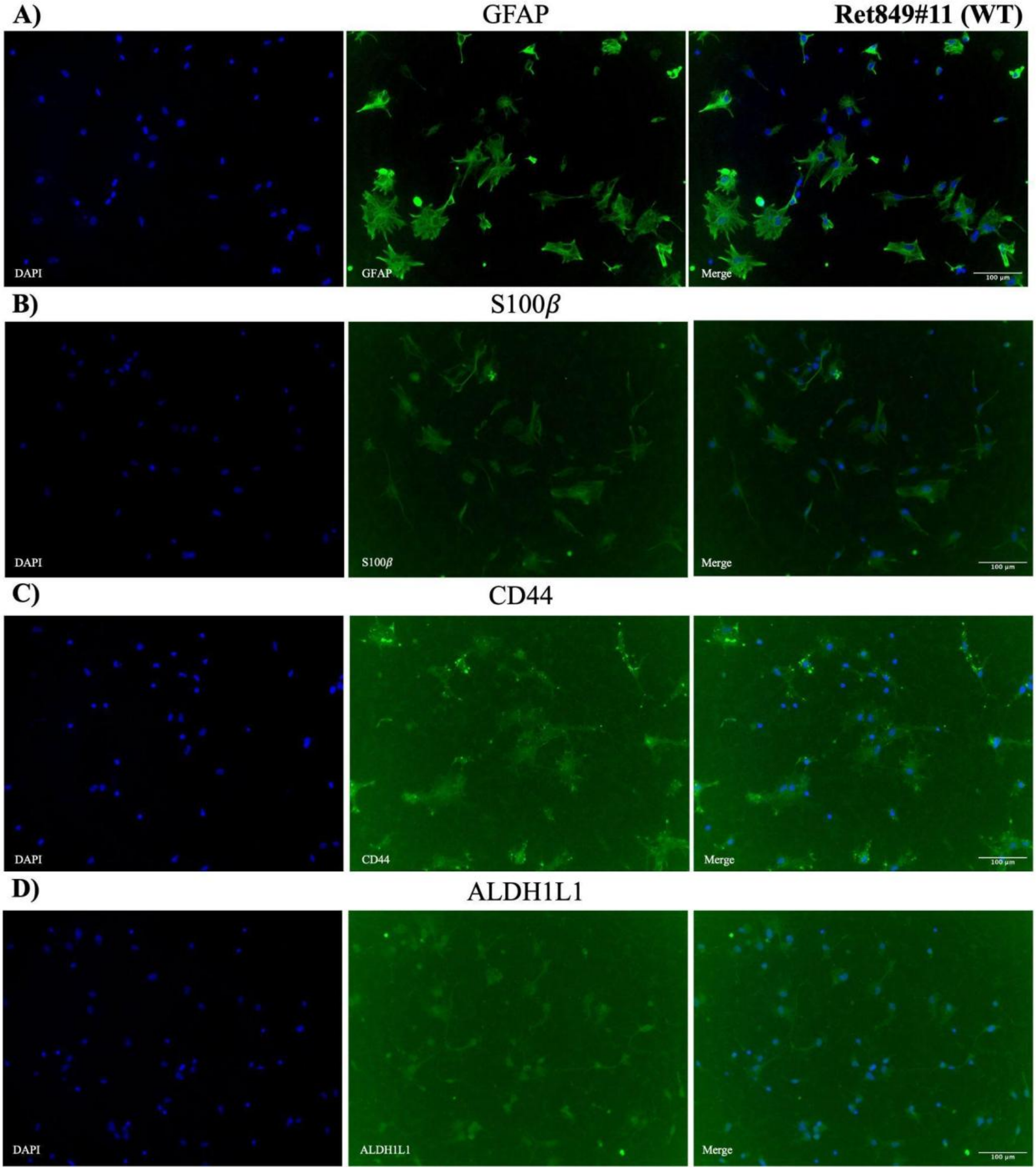

Supplemental Figure 2: Astrocyte markers in Ret849#11 mature astrocytes
Representative immunocytochemistry-immunofluorescence images of mature iPSC astrocytes. Staining shows GFAP, S100β, CD44, ALDH1L1 along with the nuclear stain DAPI. Scale bar represents 100 µm.

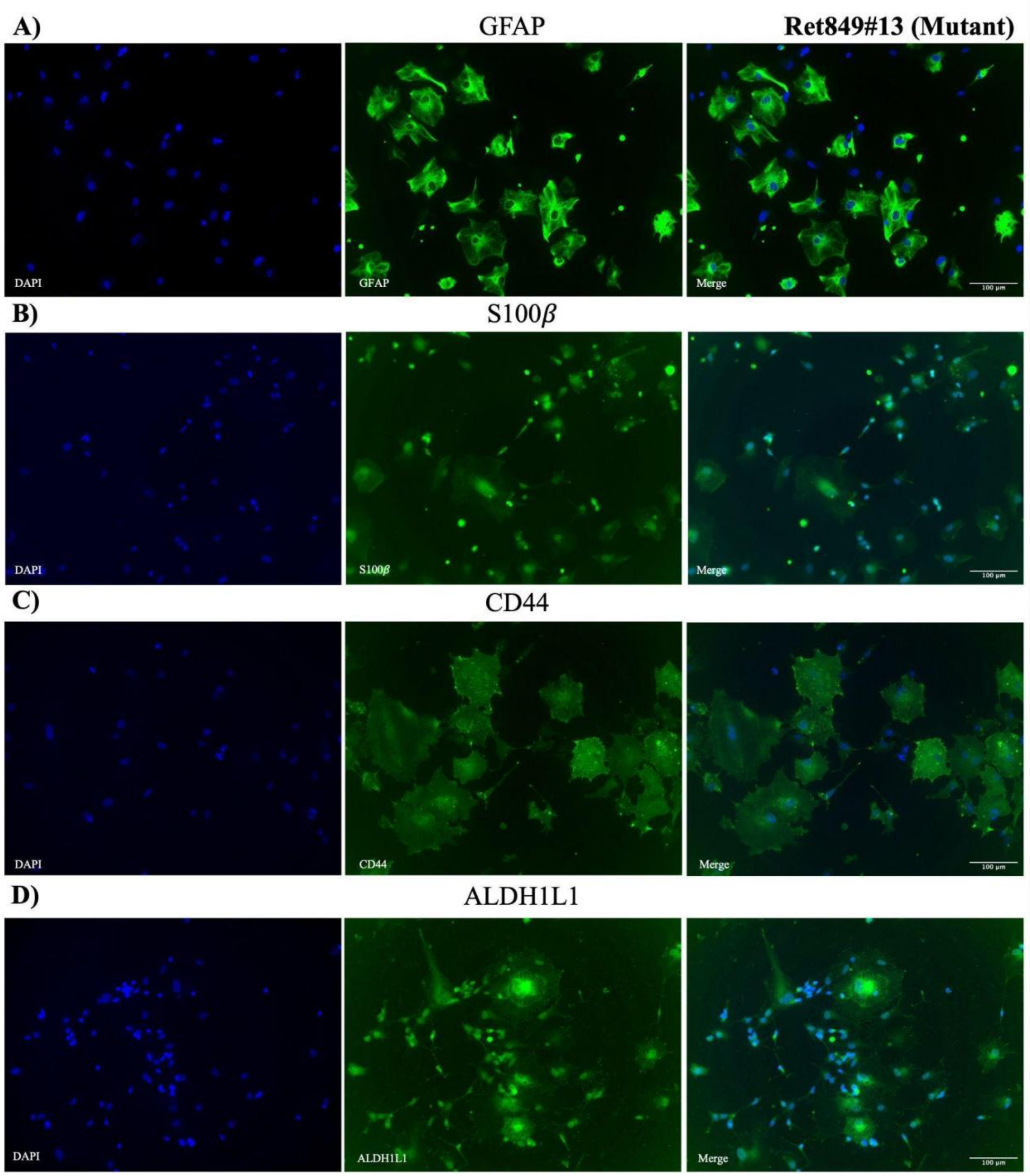

Supplemental Figure 3: Astrocyte markers in Ret849#13 mature astrocytes
Representative immunocytochemistry-immunofluorescence images of mature iPSC astrocytes. Staining shows GFAP, S100β, CD44, ALDH1L1 along with the nuclear stain DAPI. Scale bar represents 100 µm.

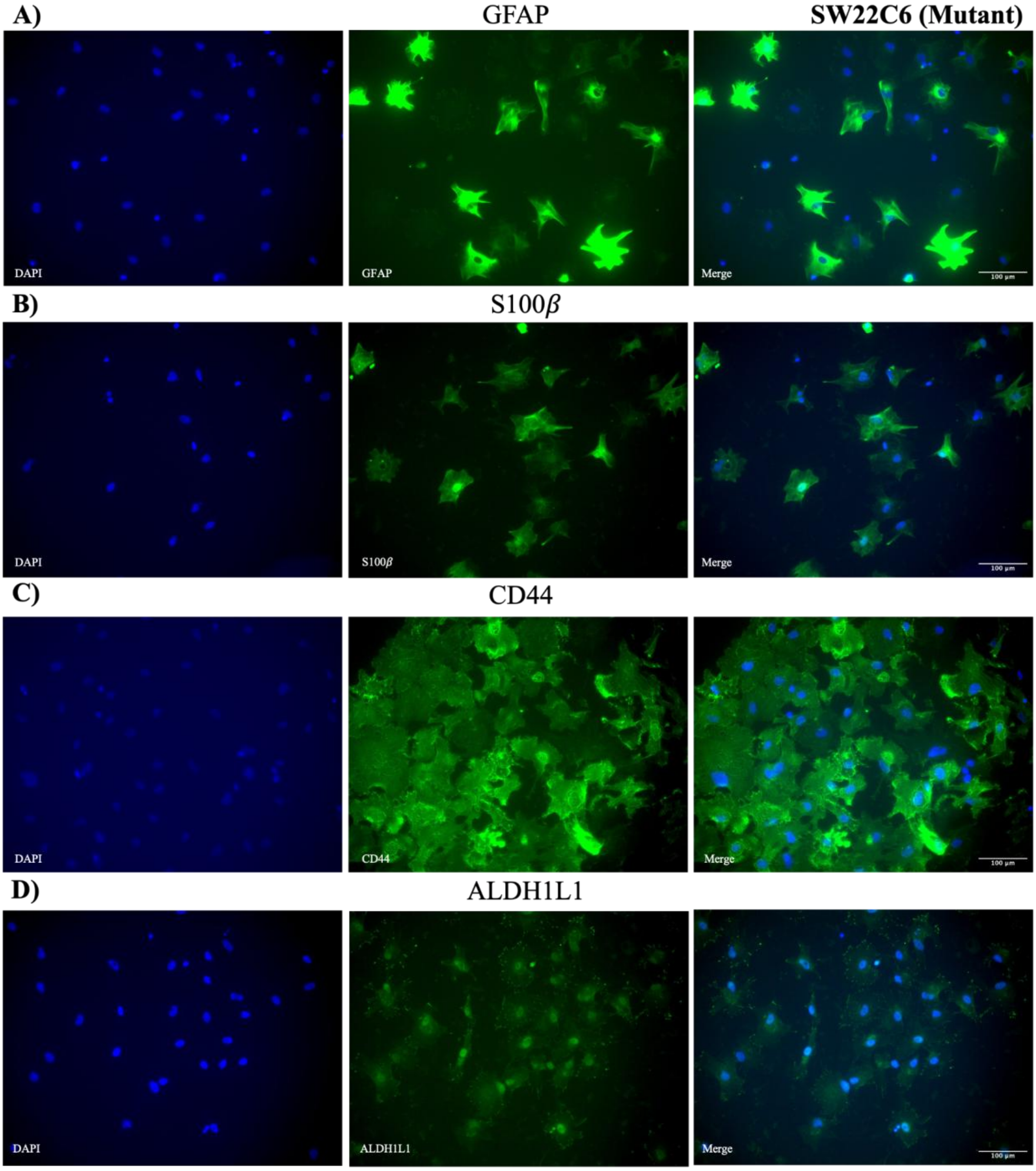

Supplemental Figure 4: Astrocyte markers in SW22C6 mature astrocytes
Representative immunocytochemistry-immunofluorescence images of mature iPSC astrocytes. Staining shows GFAP, S100β, CD44, ALDH1L1 along with the nuclear stain DAPI. Scale bar represents 100 µm.

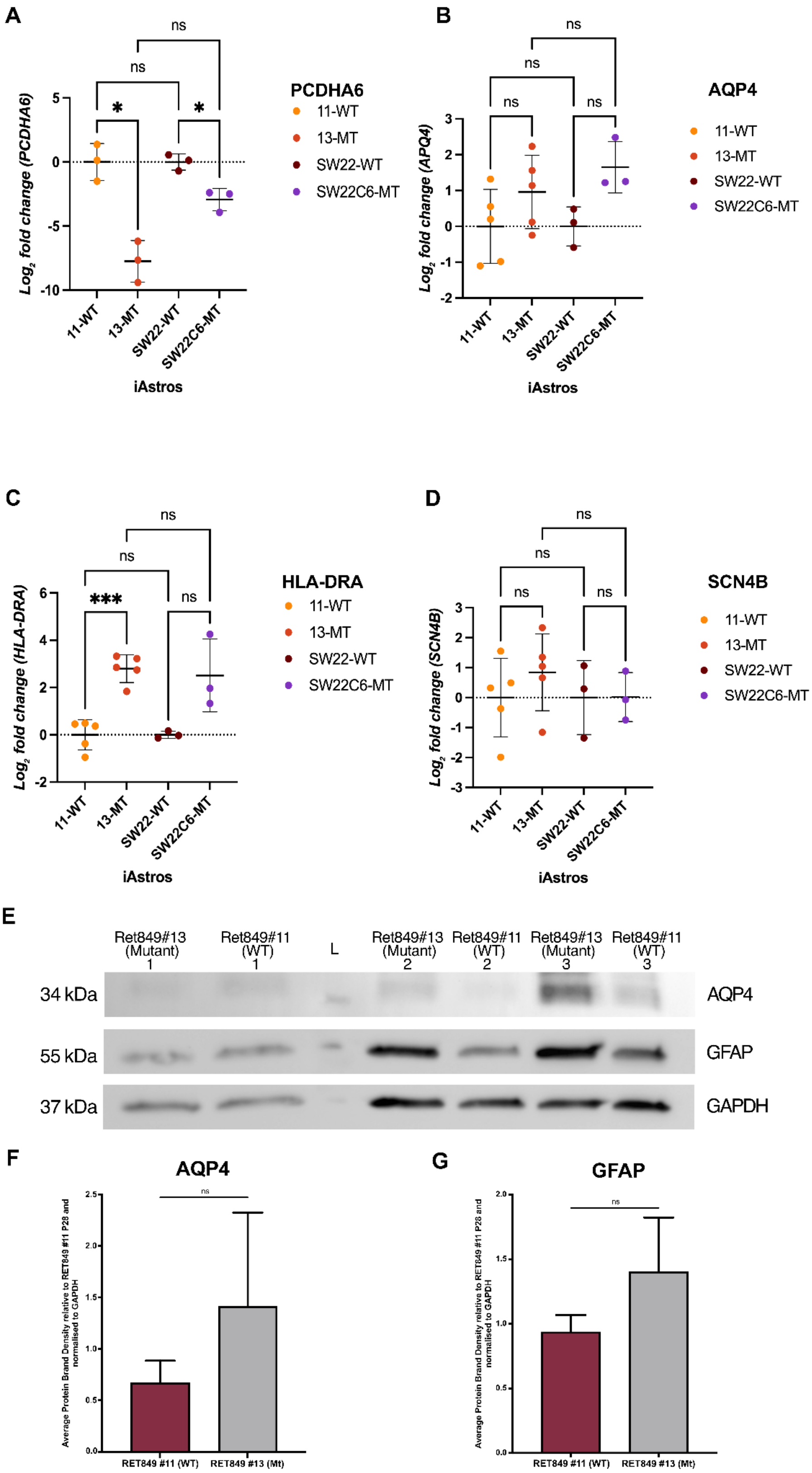

Supplemental Figure 5: Validation of RNAseq Data in mature iAstros
RT-qPCR analysis of **(A)** *PCDHA6* (downregulated in RNAseq data), **(B)** *APQ4* (upregulated in RNAseq data), **(C)** *HLA-DRA* (upregulated in RNAseq data), and **(D)** *SCN4B* (not dysregulated; negative control) in iPSC-derived astrocytes from two patient lines (13MT, SW22C6) and their isogenic controls (11WT, SW22). Expression was normalised

to *GAPDH* and is shown relative to isogenic lines (2^−ΔΔCq). Data are mean ± SD; points represent technical replicates (n = 5 for 11WT and 13MT; n = 3 for SW22 and SW22C6), each measured in technical triplicate. Log2 fold-change values were analysed by Brown–Forsythe and Welch ANOVA with Dunnett's T3 test. Patient vs isogenic comparisons are the primary analysis; cross-line comparisons indicate background variation (see Materials and Methods). ns, $P > 0.05$; *$P \leq 0.05$; ***$P \leq 0.001$. SD, standard deviation. **E)** Western blot images of staining conducted on CDKL5-null (Ret849#13) and WT (Ret849#11) mature astrocytes at three time-points in AMM (18 days, 46 days and 51 days), staining for AQP4, GFAP and GAPDH as a loading control. **F)** Semi- quantitative analysis of protein band density of AQP4, normalised to WT D18 sample. n=3 time points following AMM protocol, (1 technical replicate per time-point). Image analysis was performed using FIJI. Statistical analysis was performed in GraphPad Prism. A one-tailed paired t-test was used to determine any significant increase between WT and Cdkl5-mutant (Mt) iAstros. **G)** As for F but GFAP. A two-tailed pair t-test was used to determine whether there was any significant difference between WT and Mt given no difference was seen in transcriptomic data.

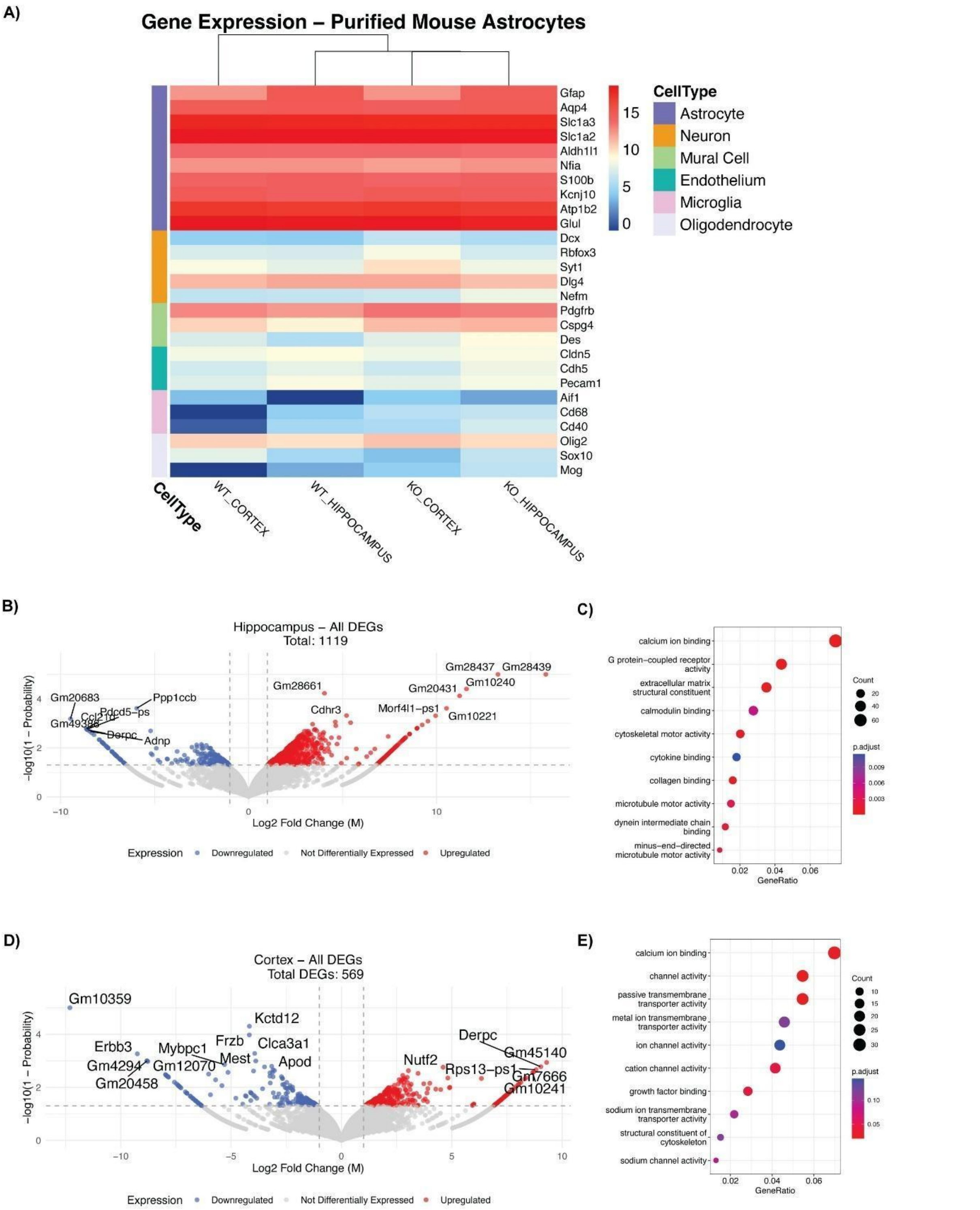

Supplemental Figure 6: RNAseq Analysis of Astrocytes purified from Cdkl5$^{-/y}$ KO and WT littermate mice.

A) Heatmap showing $\log_2$ normalised counts of genes associated with astrocytes, mural cells, endothelium, neurons, microglia and oligodendrocytes in purified Cdkl5-null (KO) and wild-

type (WT) hippocampal and cortical astrocytes. Plot generated in RStudio using pheatmaps package with RColorBrewer.
B) Volcano plot showing -log10(1-probability) against log_2 fold change (lfc) differentially expressed genes in KO mouse hippocampal astrocytes compared to WT. Each dot represents a gene, coloured dots represent significantly differentially expressed genes, up- or downregulated according to the legend below each plot. Lfc threshold > 1, probability threshold > 0.95. Dashed line indicates location of significance threshold.
C) Top 10 most significantly enriched Molecular Function Gene Ontology annotations for mouse hippocampus DEGs.
D) As for B but Cortex.
E) As for C but for Cortex.

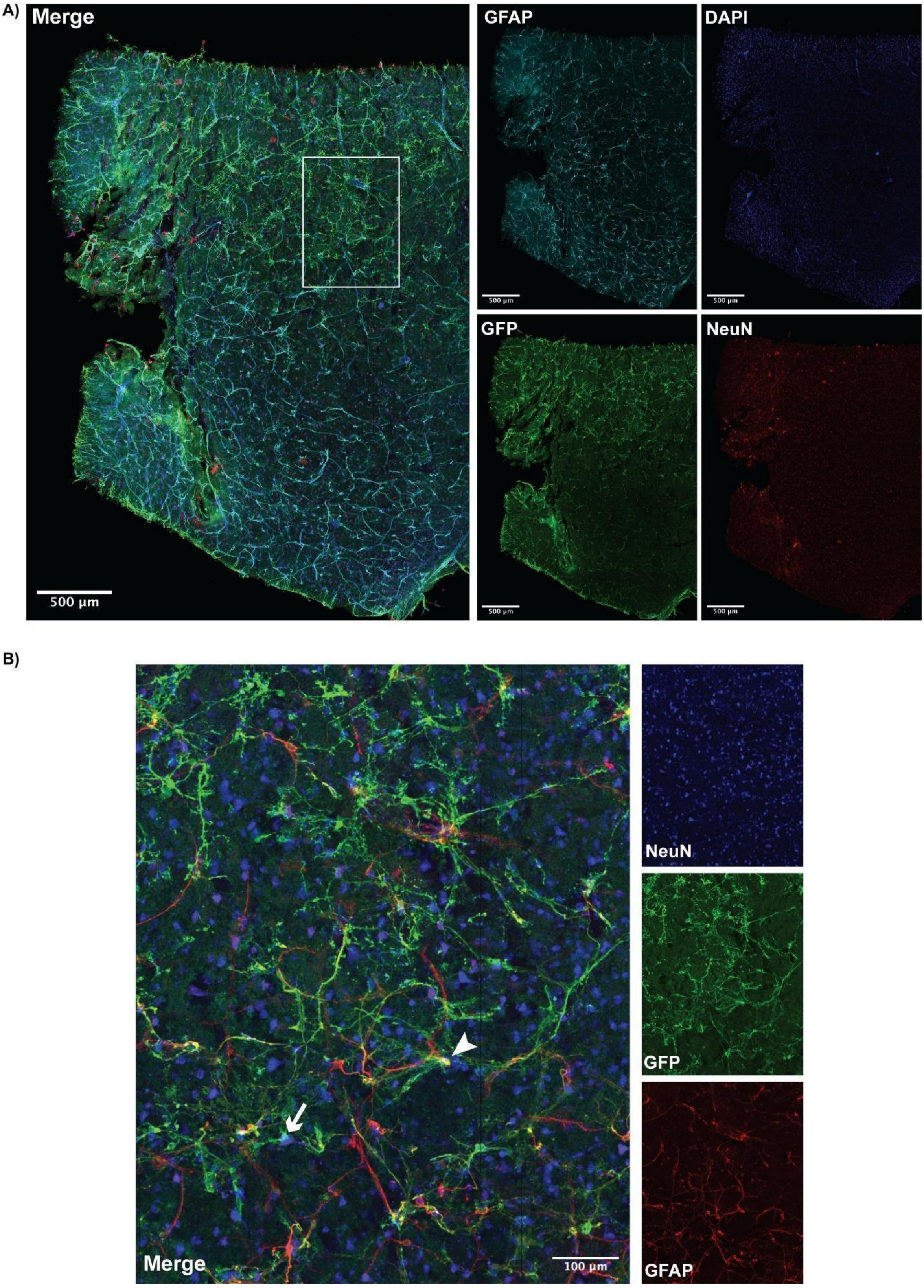
A)
Merge
500 µm
GFAP
500 µm
DAPI
500 µm
GFP
500 µm
NeuN
500 µm
B)
Merge
100 µm
NeuN
GFP
GFAP

Supplemental Figure 7: GFP Expression in adult human organotypic slice transduced with Scrambled shRNA.

A) Representative confocal scanning images of human adult cortical slice transduced with AAVDJ-U6-ScrambledshRNA-CMV-eGFP (Scrambled shRNA), and stained with GFAP (cyan), GFP (green), NeuN (red) and the nuclear marker DAPI (blue). Scale bars represent 500 µm. Images were taken using a Zeiss LSM800 Airyscan confocal microscope.

B) Zoomed in area of A), position indicated by white box in A). In this image, DAPI is removed for easier visualisation: NeuN is blue, GFP is still green and GFAP is red. Scale bar represents 100 µm. Arrow shows GFP colocalization with NeuN (cyan, neuronal transduction) whilst pointed triangle shows GFP colocalization with GFAP (yellow, astrocytic transduction).

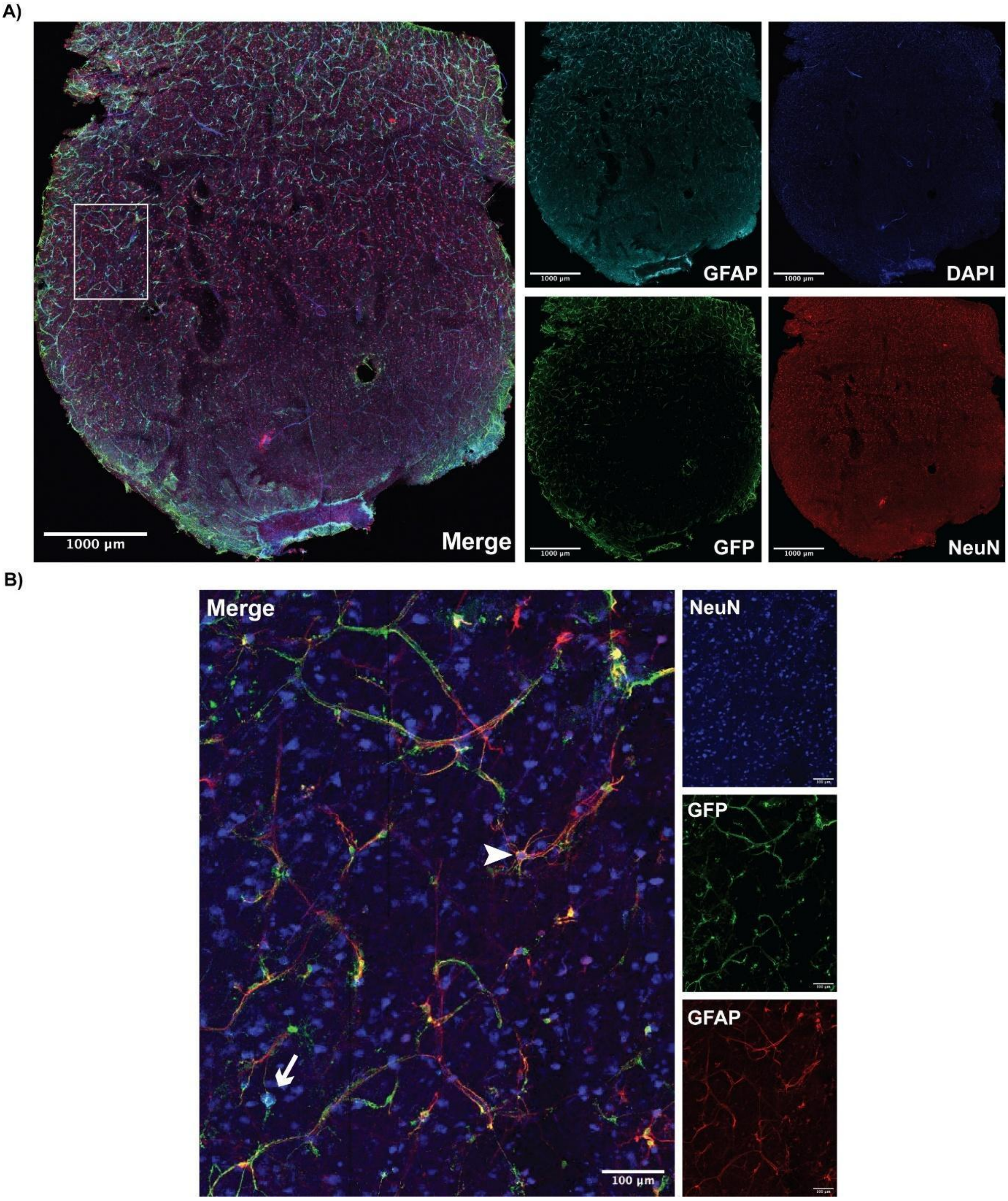

Supplemental Figure 8: GFP Expression in human organotypic slices transduced with ShAAV. A) Representative confocal scanning images of human adult cortical slice transduced with AAVDJ-U6-CDKL5shRNA-CMV-eGFP (CDKL5 shRNA) and stained with GFAP (cyan), GFP (green), NeuN (red) and the nuclear marker DAPI (blue). Scale bars represent 500 µm. Images were taken using a Zeiss LSM800 Airyscan confocal microscope.

B) Zoomed in area of A), position indicated by white box in A). In this image, DAPI is removed for easier visualisation: NeuN is blue, GFP is still green and GFAP is red. Scale bar represents 100 µm. Arrow shows GFP colocalization with NeuN (cyan, neuronal transduction) and pointed triangle shows GFP colocalization with GFAP (yellow, astrocytic transduction).

Supplemental full-sized Western Blot images

A)

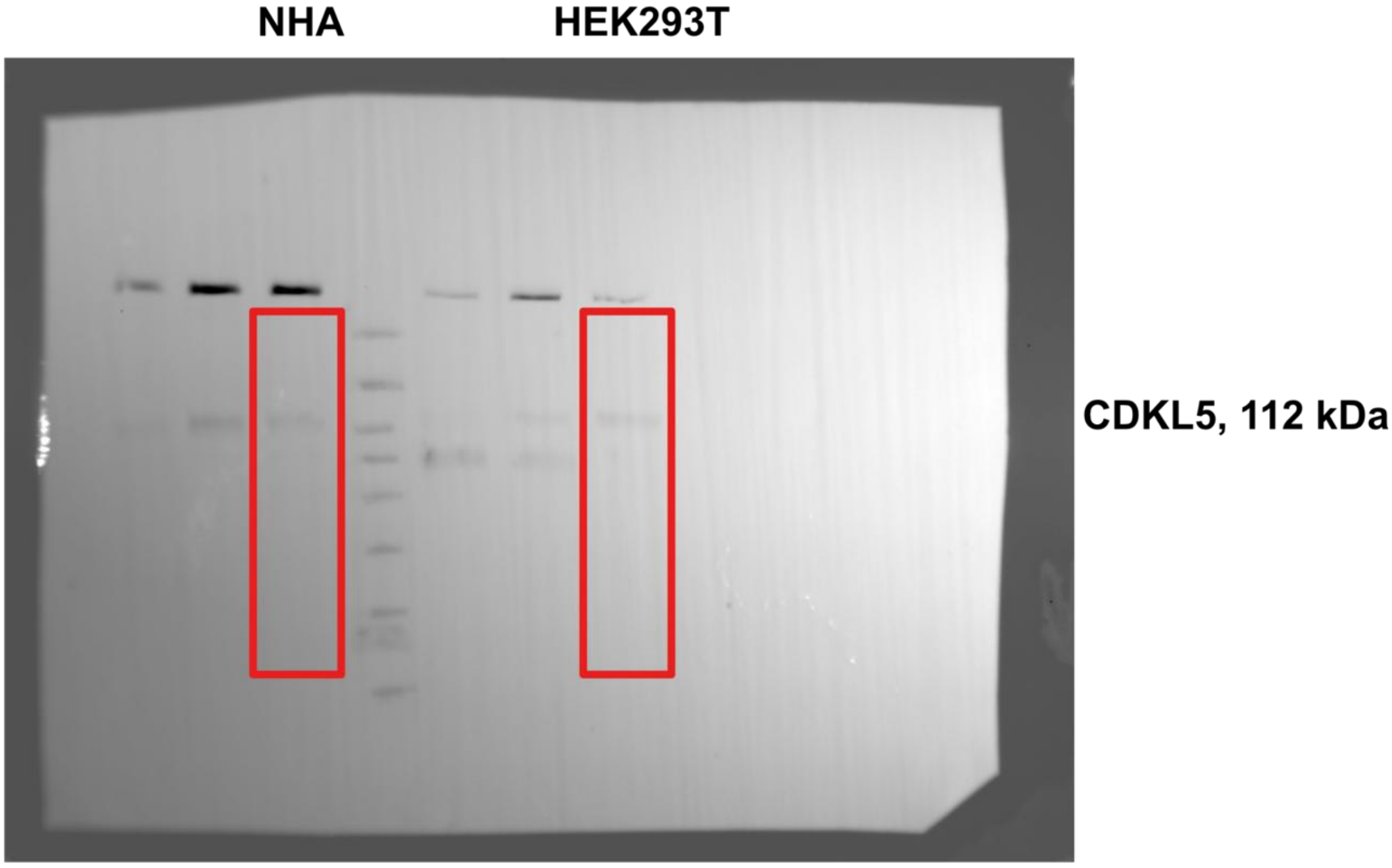

B)

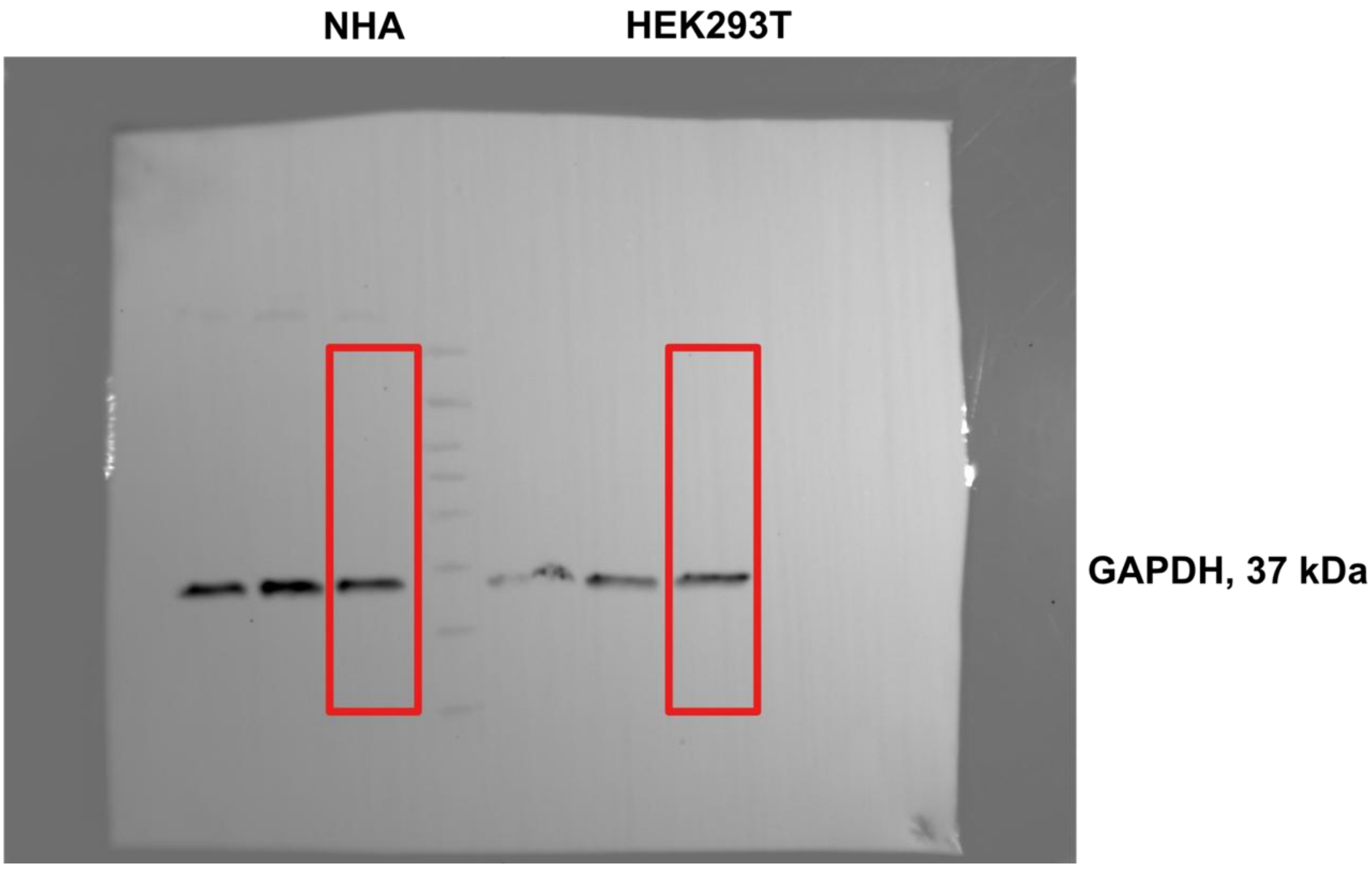

Supplemental Figure 9: Full sized original membrane images of western blot shown in Figure 1A. Red box highlights lanes used in main figure. Unlabelled lanes are not relevant and not included in the manuscript. Membrane was probed for **A)** CDKL5 and **B)** GAPDH. N.B. The membrane was cut on right-hand-side after first probe to allow for staining in a smaller container. No lanes were cut off.

A)

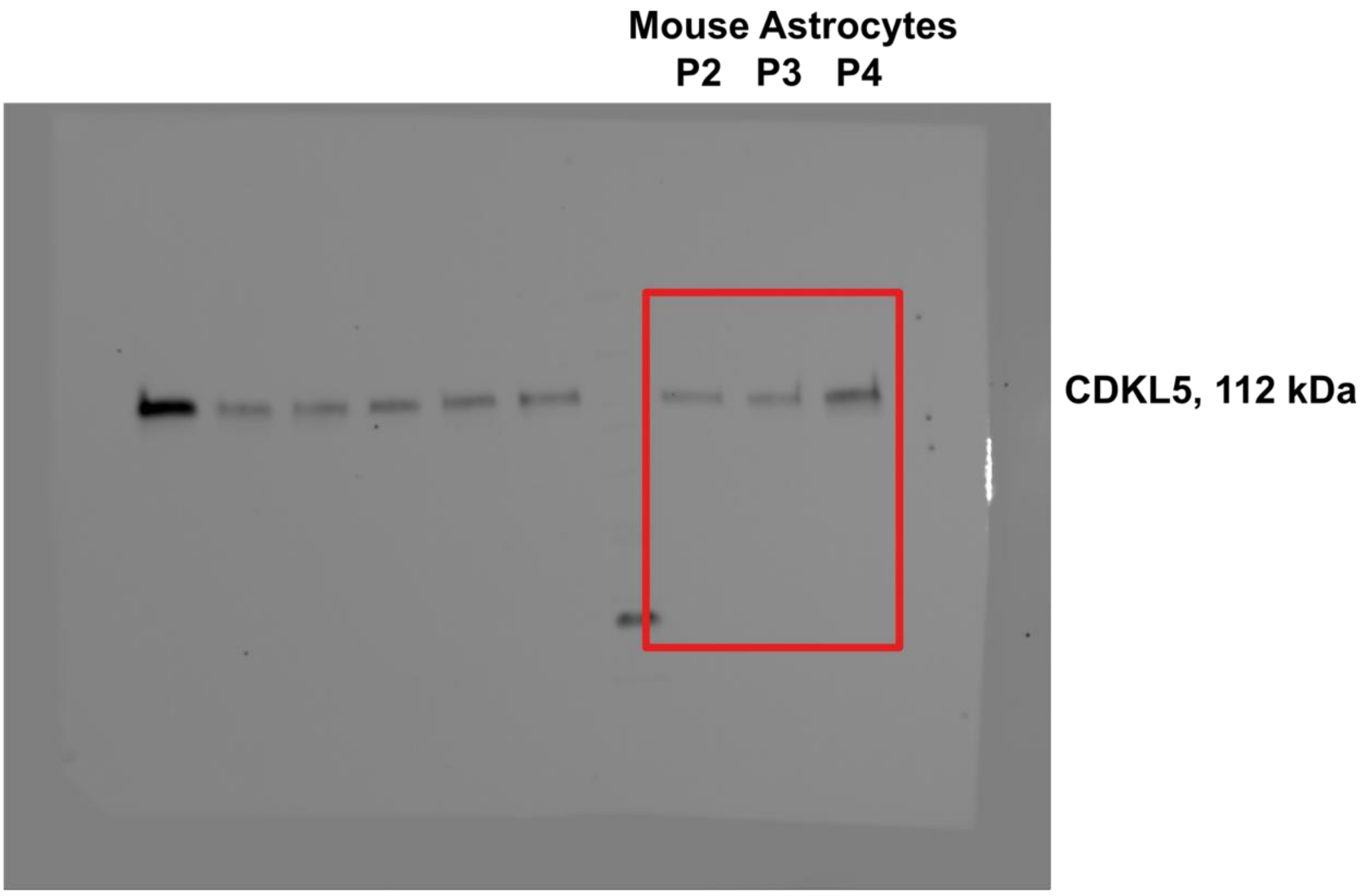
Mouse Astrocytes
P2 P3 P4
CDKL5, 112 kDa

B)

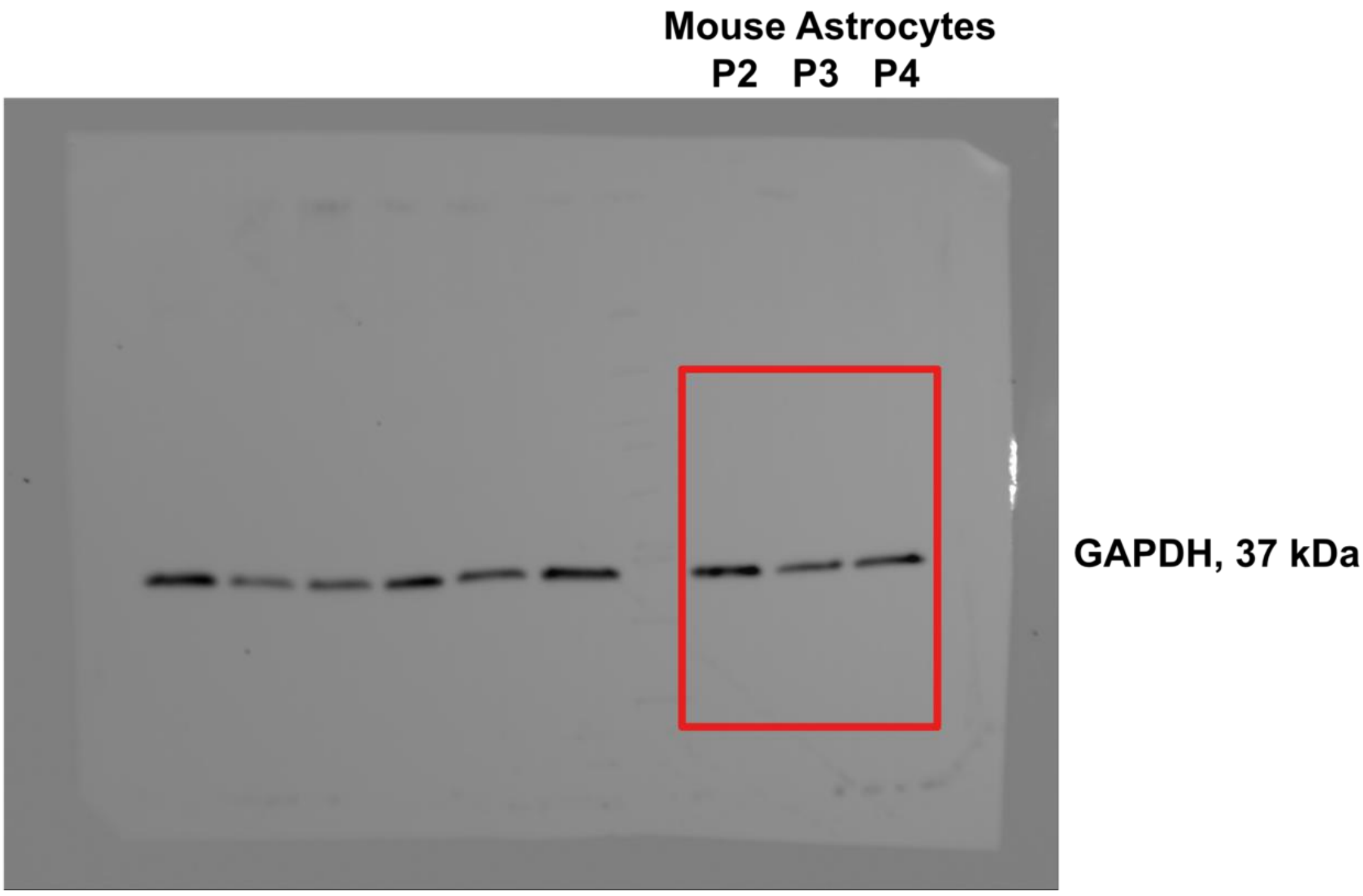
Mouse Astrocytes
P2 P3 P4
GAPDH, 37 kDa

Supplemental Figure 10: Full sized original membrane images of western blot shown in Figure 1B. Red box highlights lanes used in main figure. Unlabelled lanes are not relevant and not included in the manuscript. Membrane was probed for **A)** CDKL5 and **B)** GAPDH.

A)

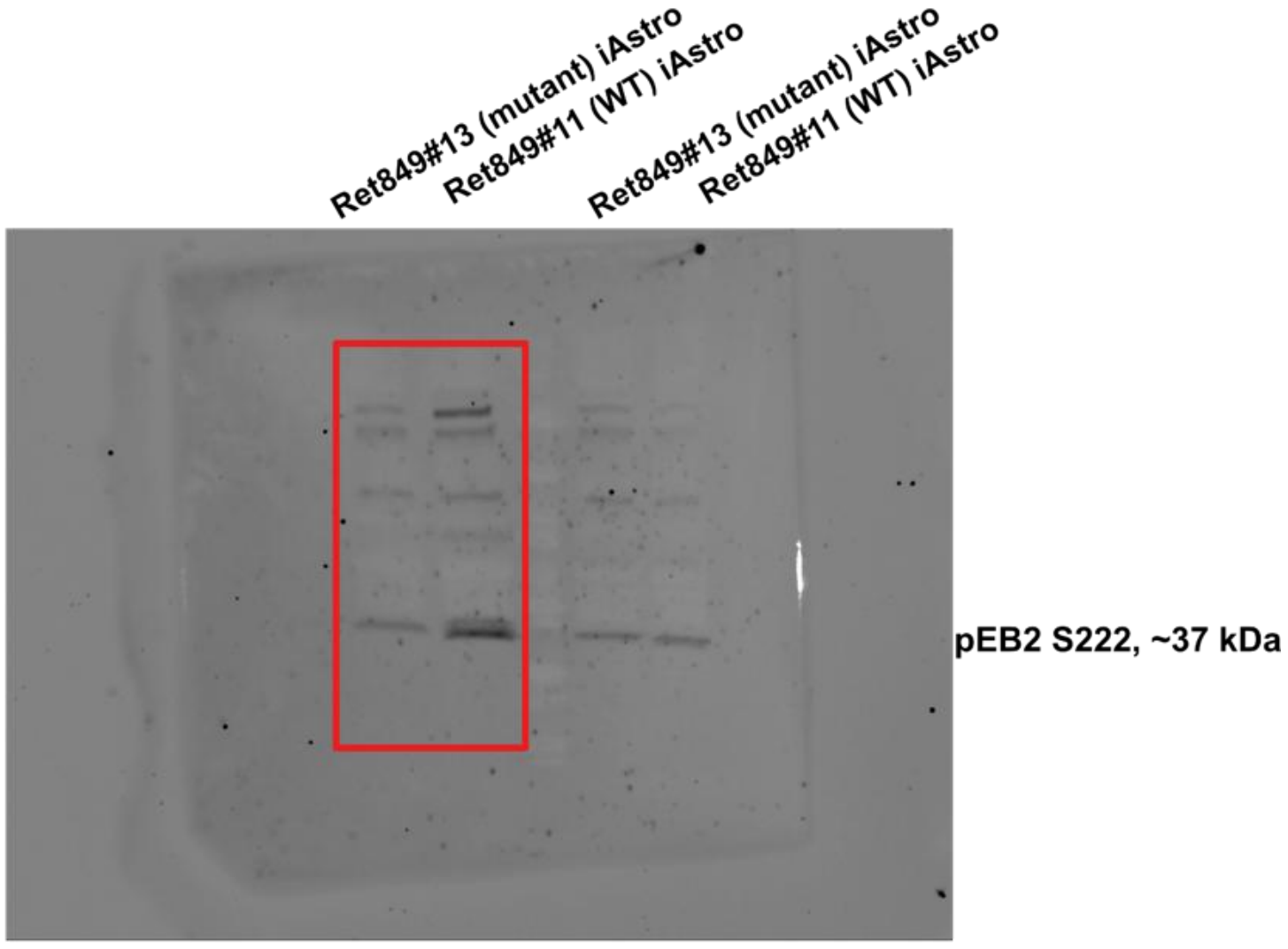

B)

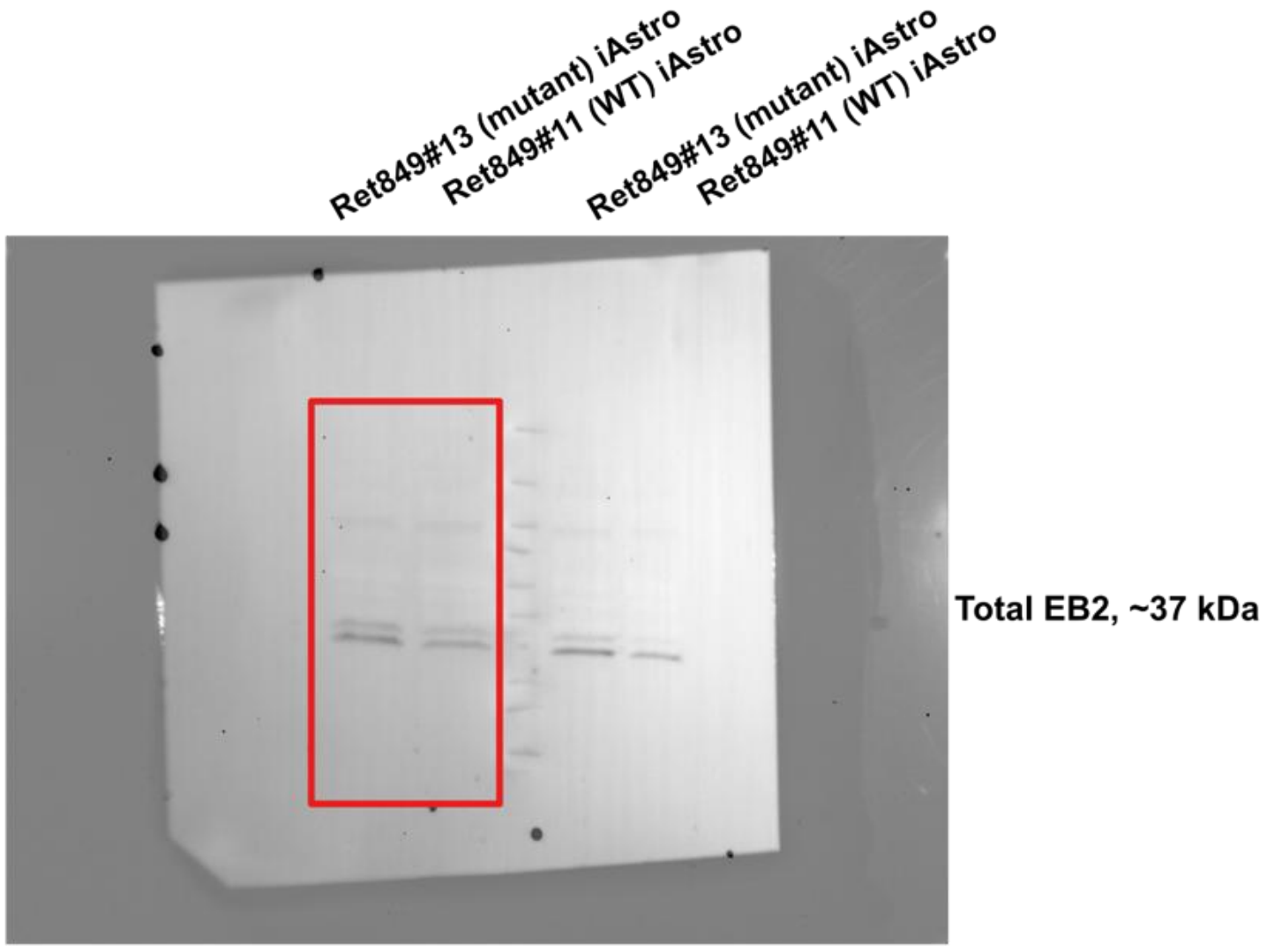

C)

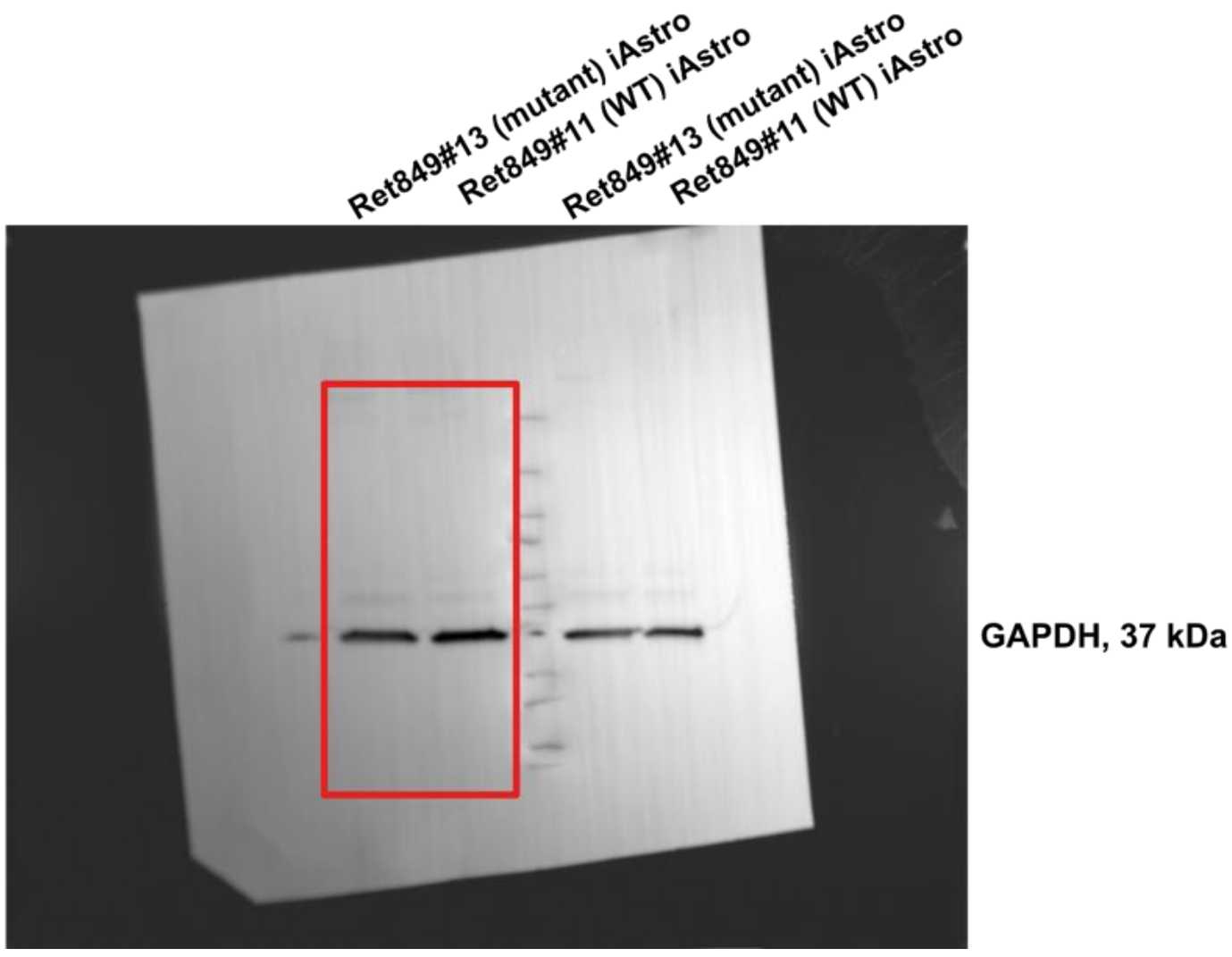

Supplemental Figure 11: Full sized original membrane images of western blot shown in Figure 4D. Red box highlights lanes used in main figure. Membrane was probed for **A)** pEB2; **B)** Total EB2 and **C)** GAPDH.

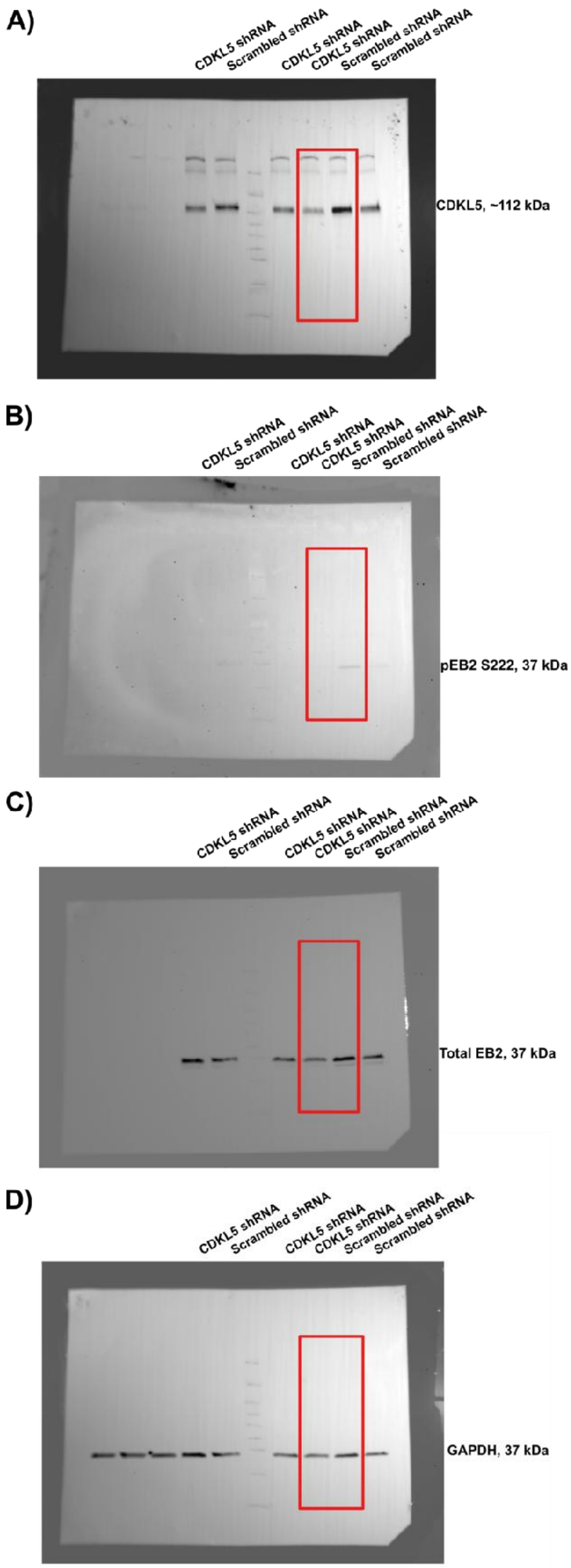

Supplemental Figure 12: Full sized original membrane images of western blot shown in Figure 5B. Red box highlights lanes used in main figure. Unlabelled lanes are not relevant

and not included in the manuscript. Membrane was probed for **A)** CDKL5**; B)** pEB2; **C)** Total EB2 and **D)** GAPDH.

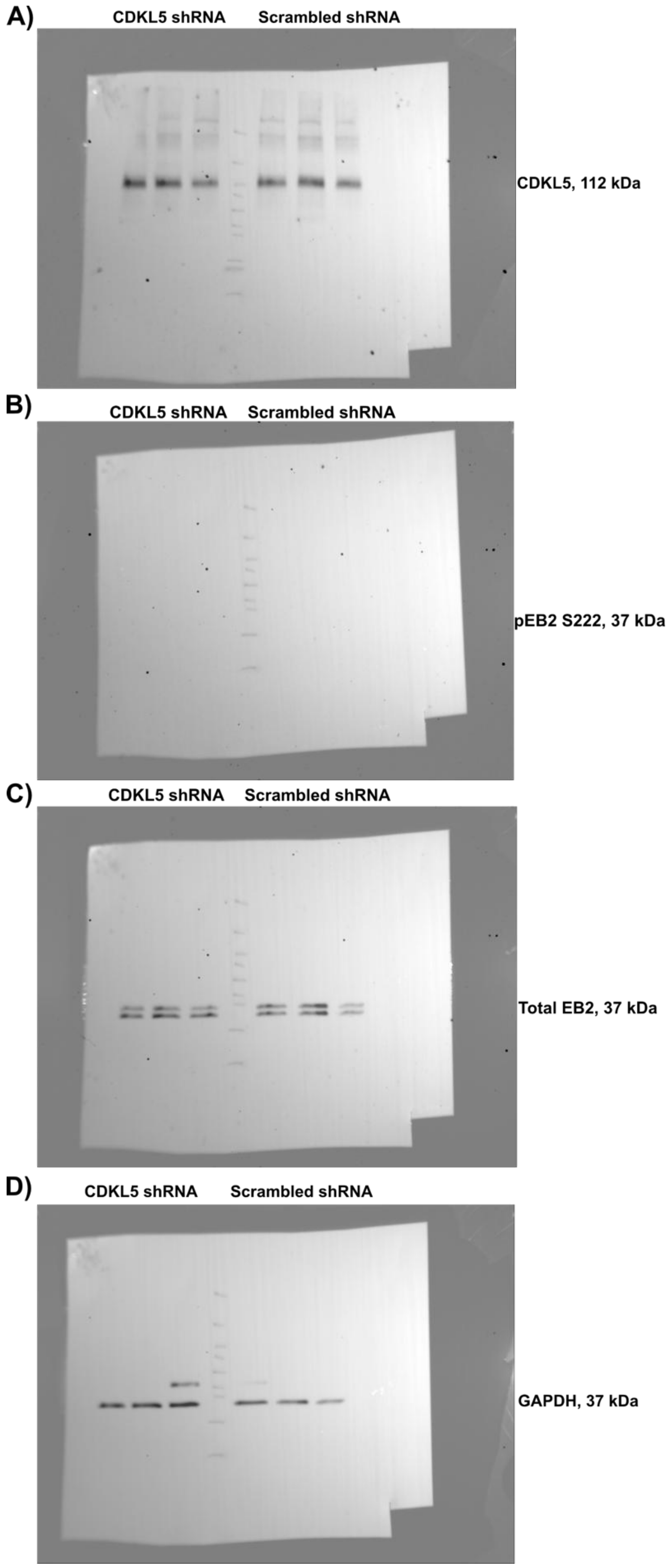

Supplemental Figure 13: Full sized original membrane images of western blot shown in Figure 5F. Lanes 1-3 are adult organotypic brain slices treated with AAV encoding CDKL5

shRNA, lane 4 is the protein ladder, lanes 4-6 is slices treated with AAV encoding the scrambled shRNA sequence. Membrane was probed for **A)** CDKL5**; B)** pEB2; **C)** Total EB2 and **D)** GAPDH.

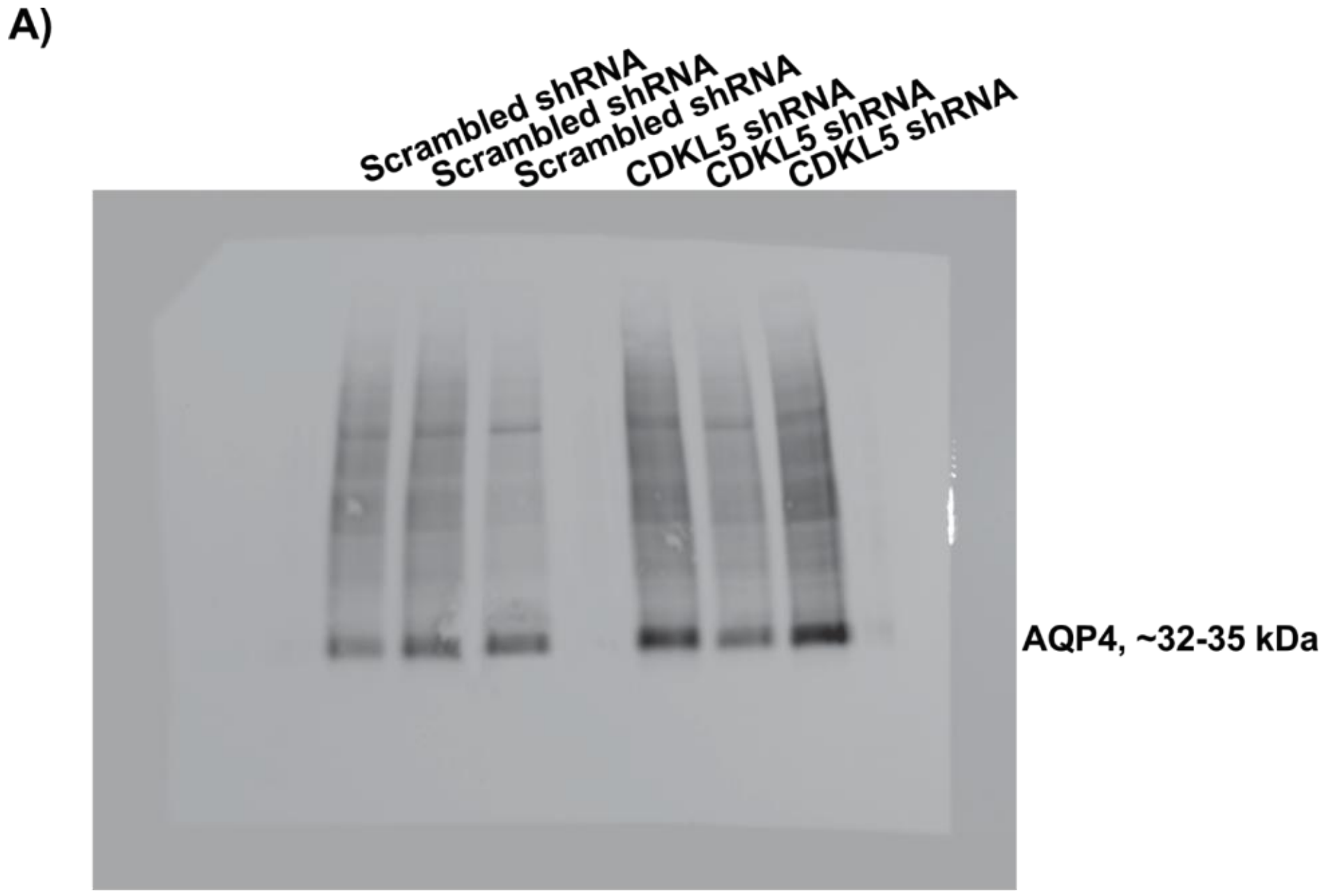

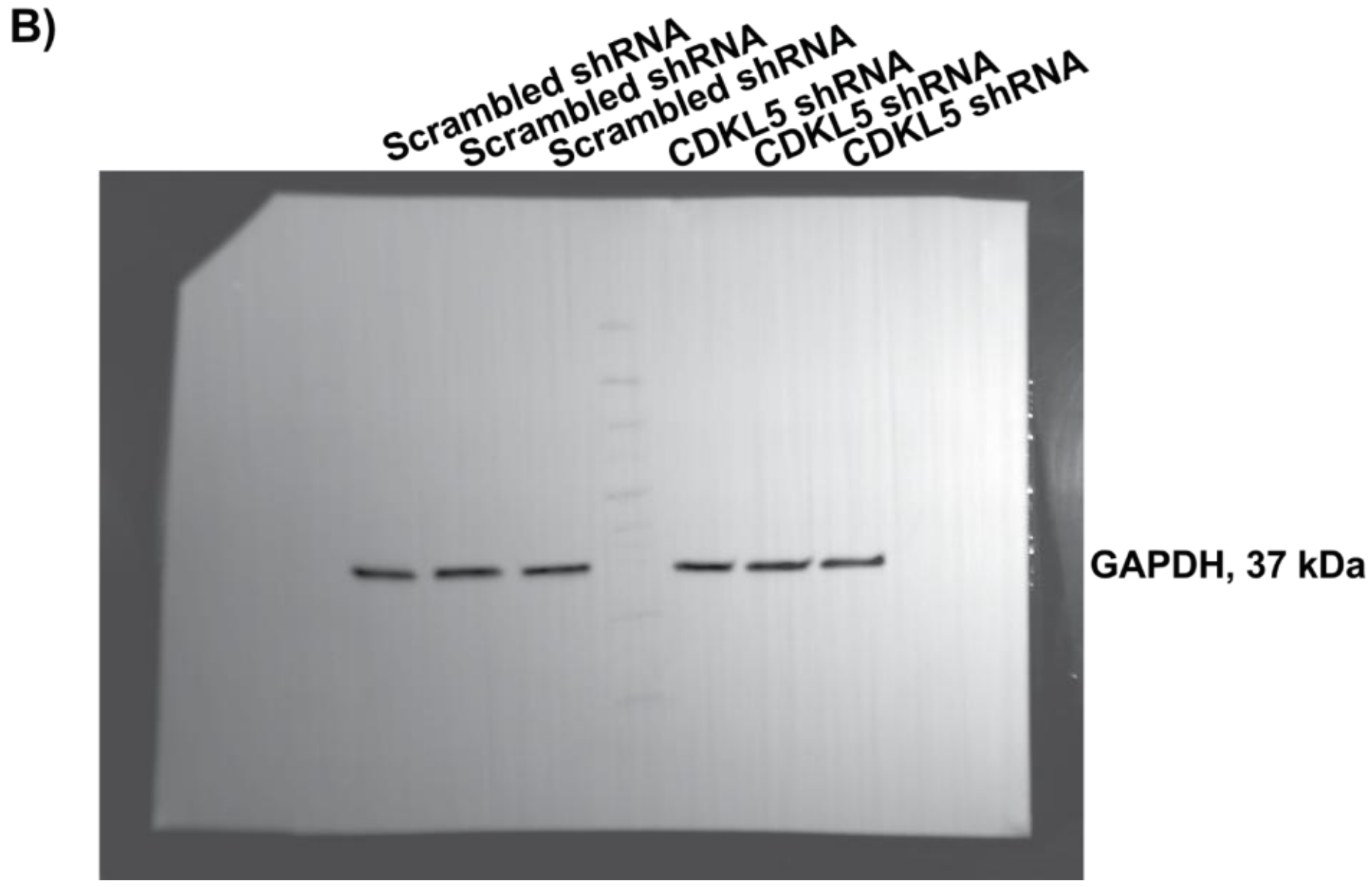

Supplemental Figure 14: Full sized original membrane images of western blot shown in Figure 7F. Membrane was probed for **A)** AQP4**; B)** GAPDH.

A)

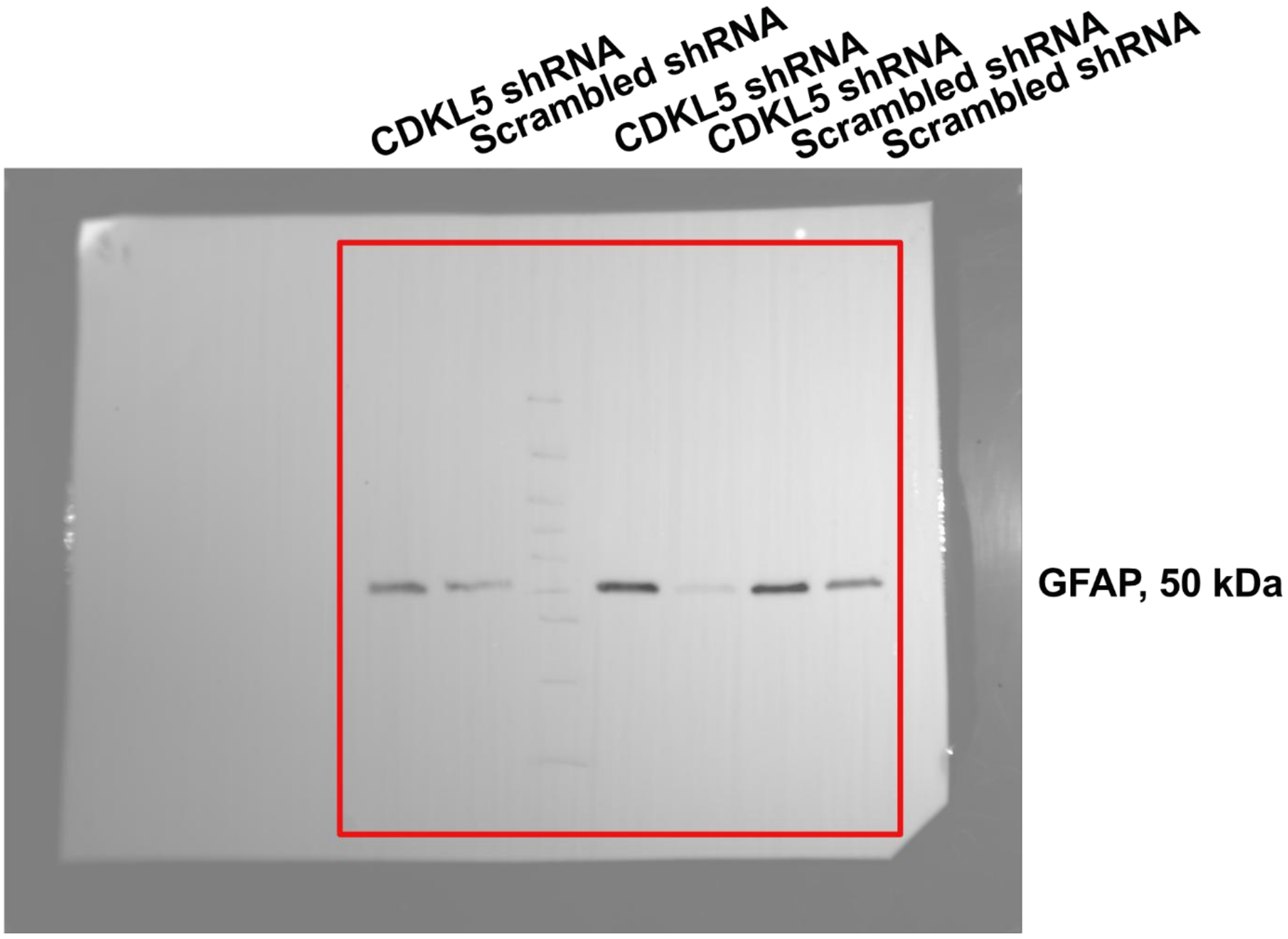

B)

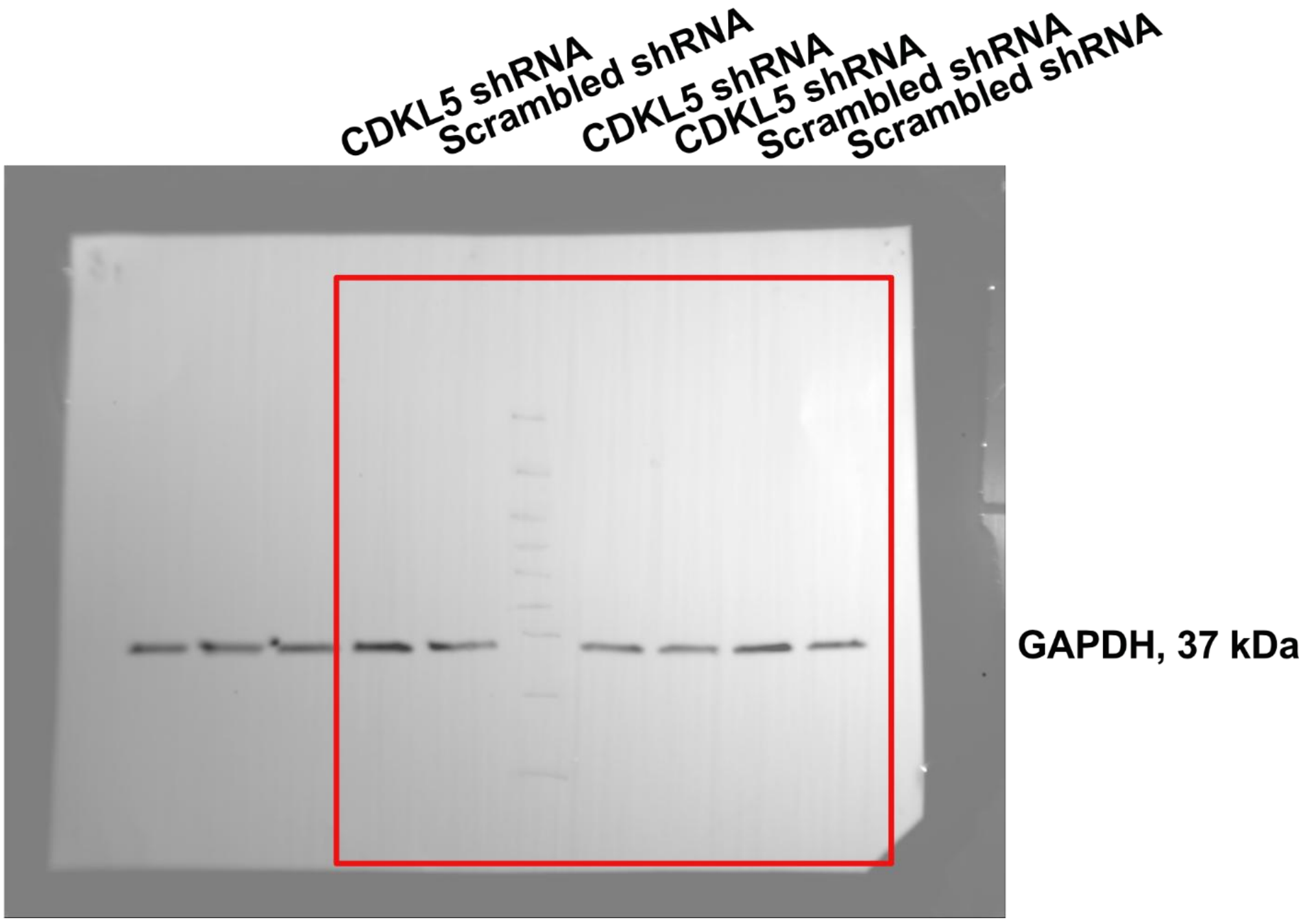

Supplemental Figure 15: Full sized original membrane images of western blot shown in Figure 7G. Red box highlights lanes used in main figure. Unlabelled lanes are not relevant and not included in the manuscript. Membrane was probed for **A)** GFAP and **B)** GAPDH.

A)

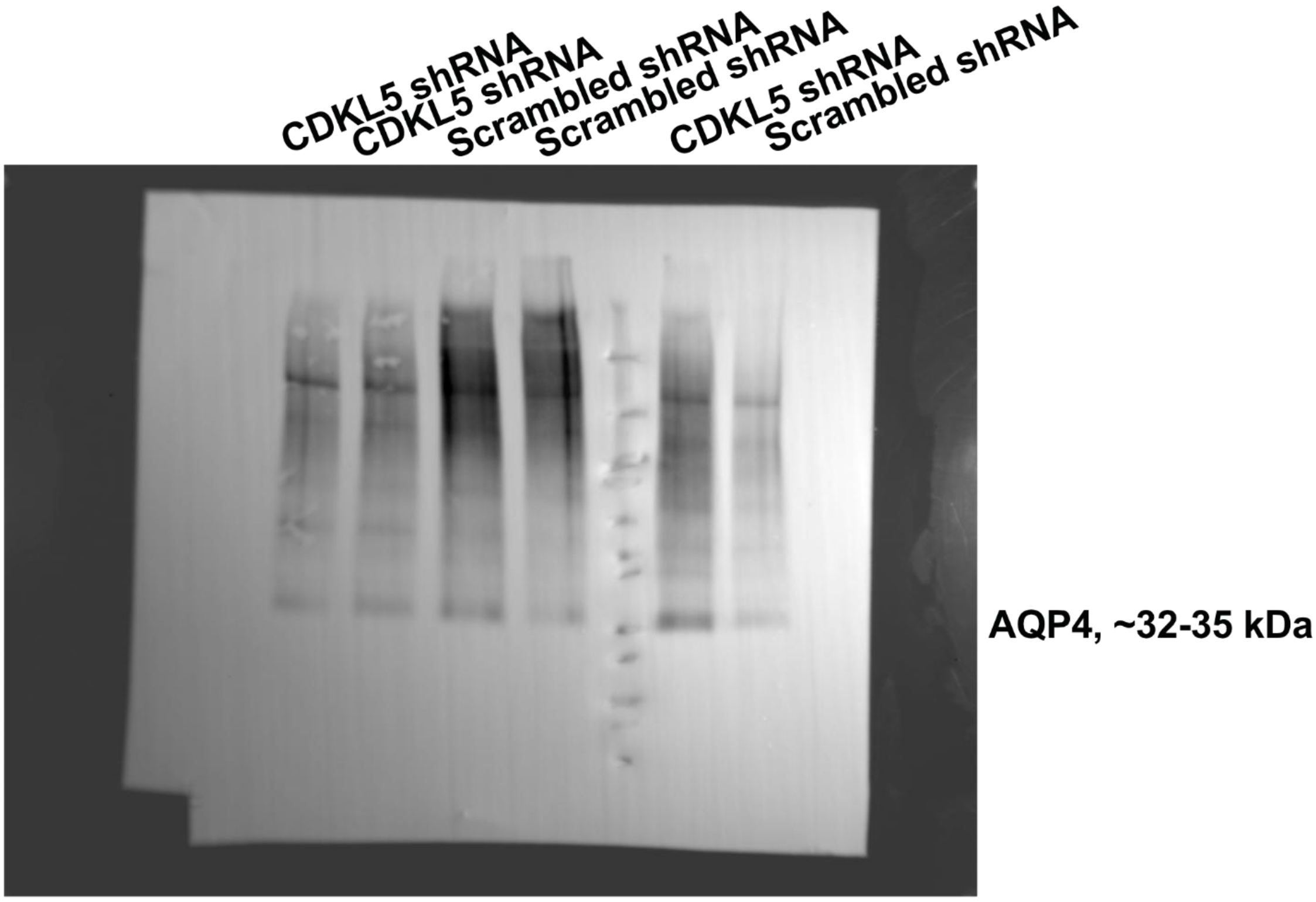

B)

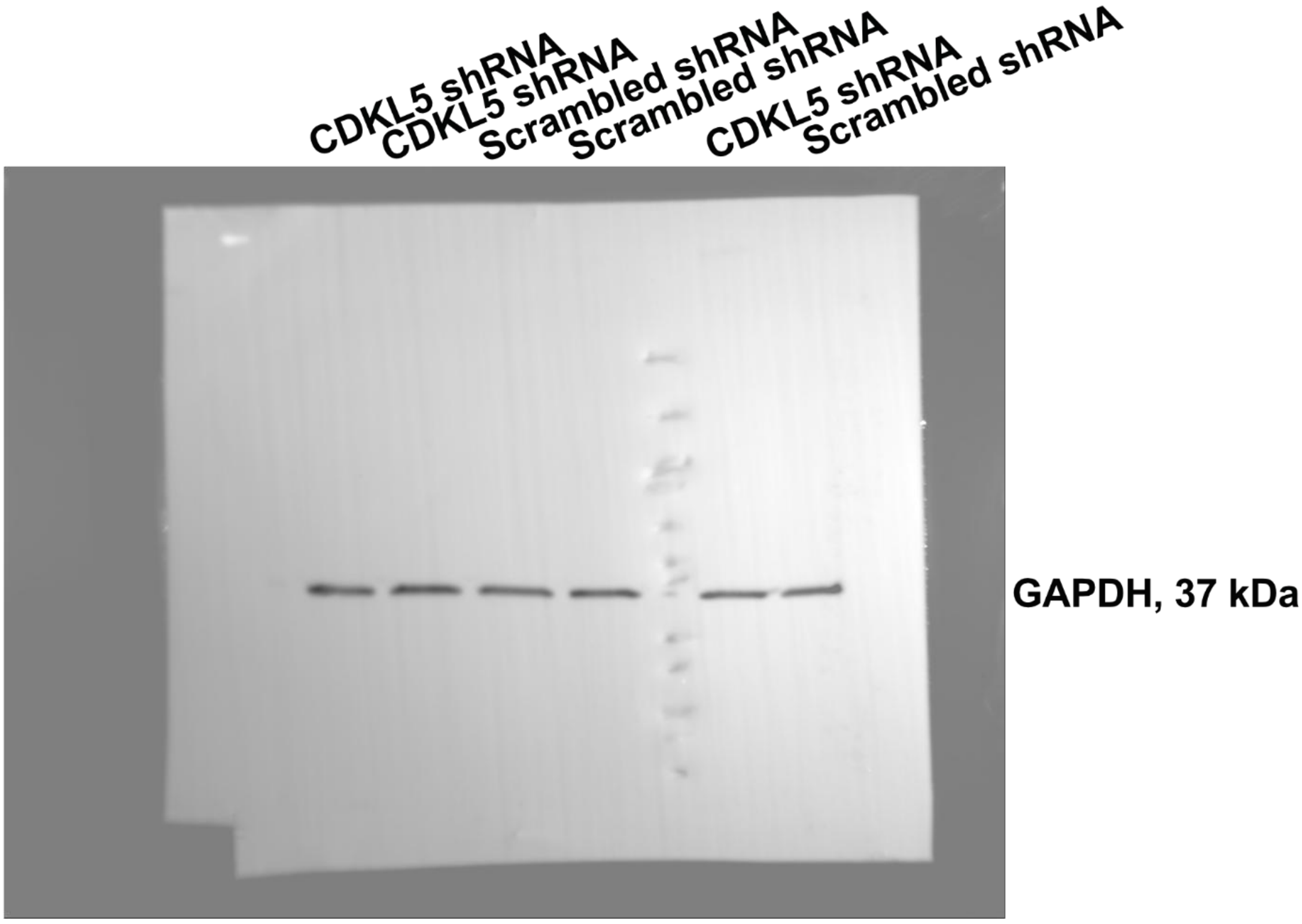

Supplemental Figure 16: Full sized original membrane images of western blot shown in Figure 7J. Membrane was probed for **A)** AQP4**; B)** GAPDH.

A)

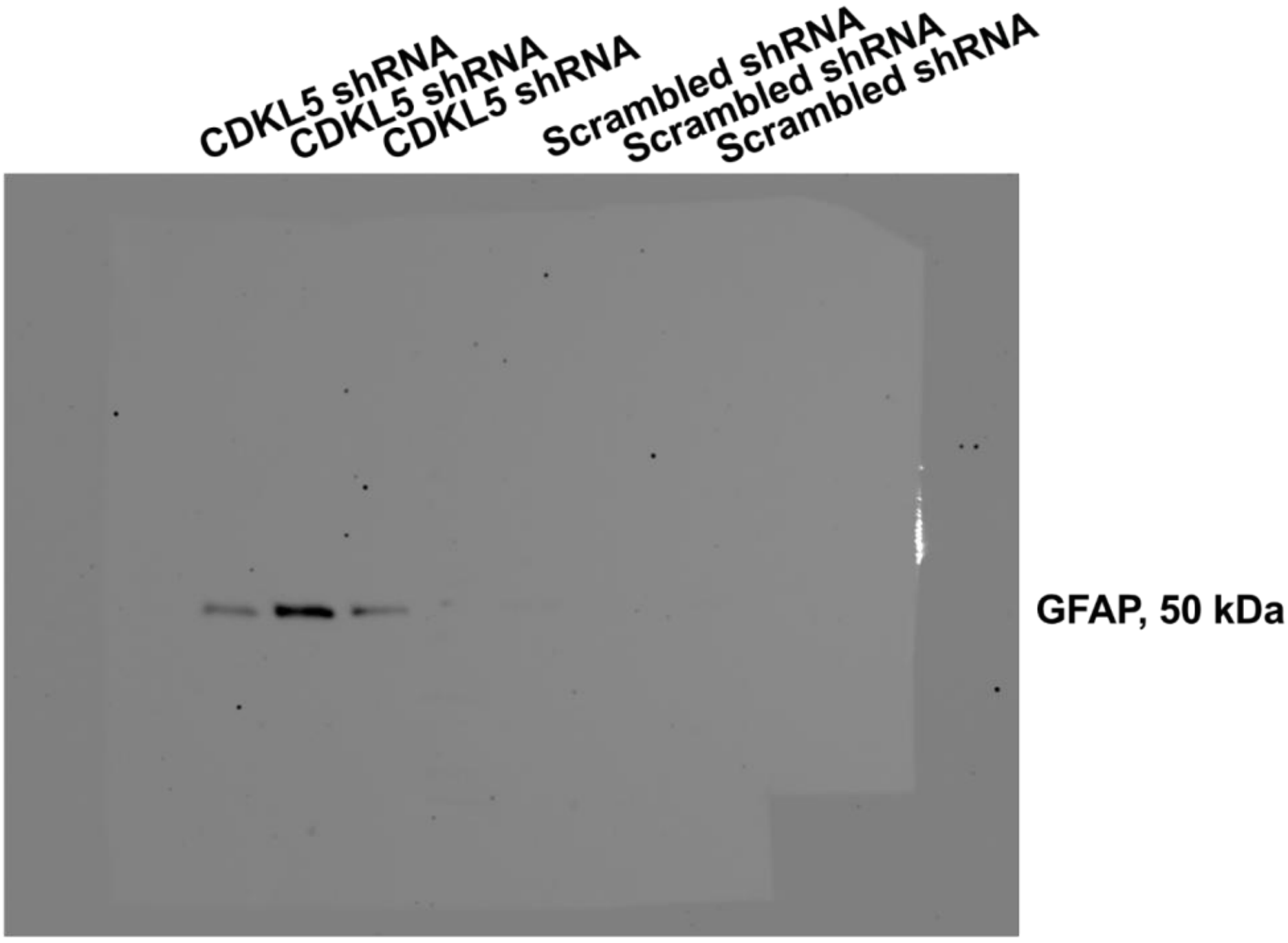

B)

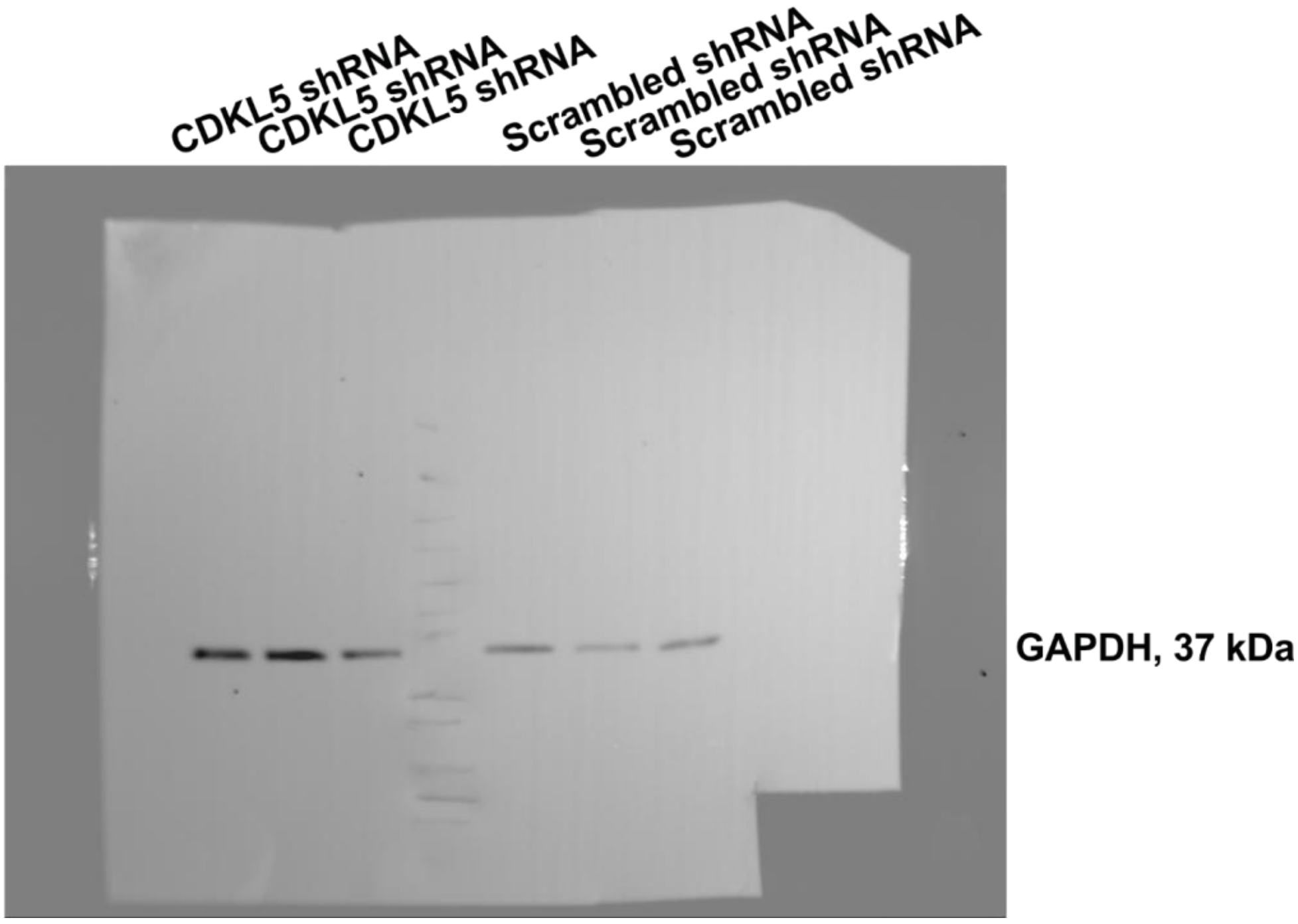

Supplemental Figure 17: Full sized original membrane images of western blot shown in Figure 7K. Membrane was probed for **A)** GFAP; **B)** GAPDH.

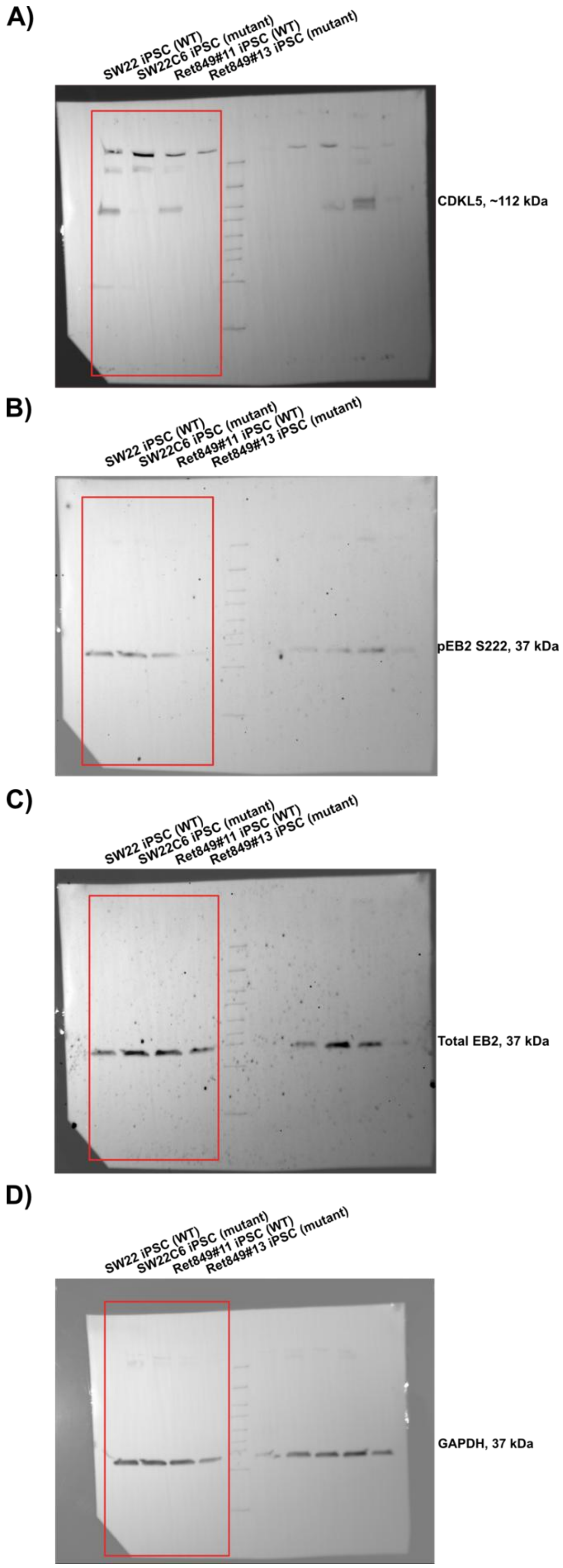

Supplemental Figure 18: Full sized original membrane images of western blot shown in Supplementary Figure 1B. Red box highlights lanes used in main figure. Unlabelled lanes

are not relevant and not included in the manuscript. Membrane was probed for **A)** CDKL5**;** **B)** pEB2; **C)** Total EB2 and **D)** GAPDH.

A)

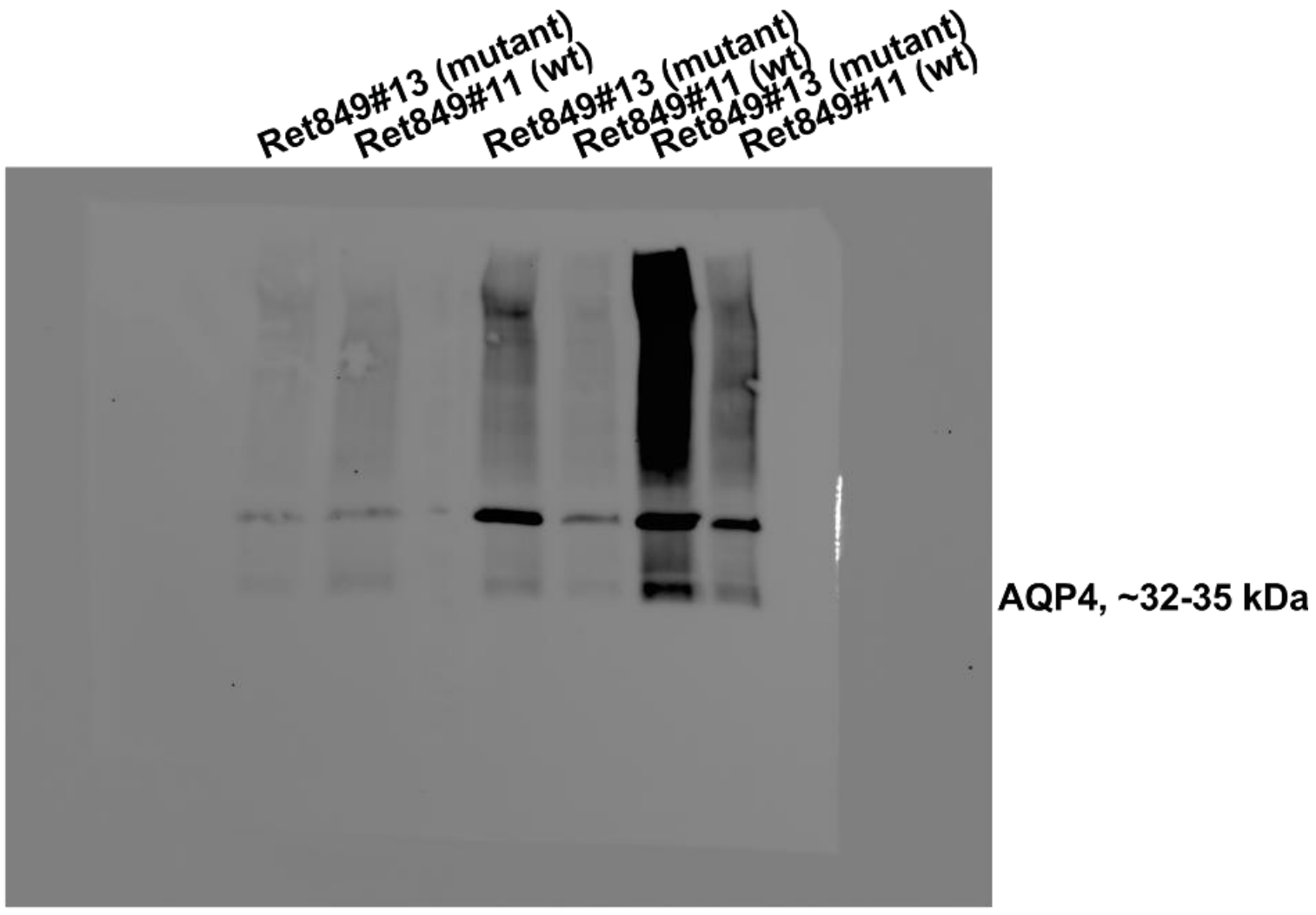

B)

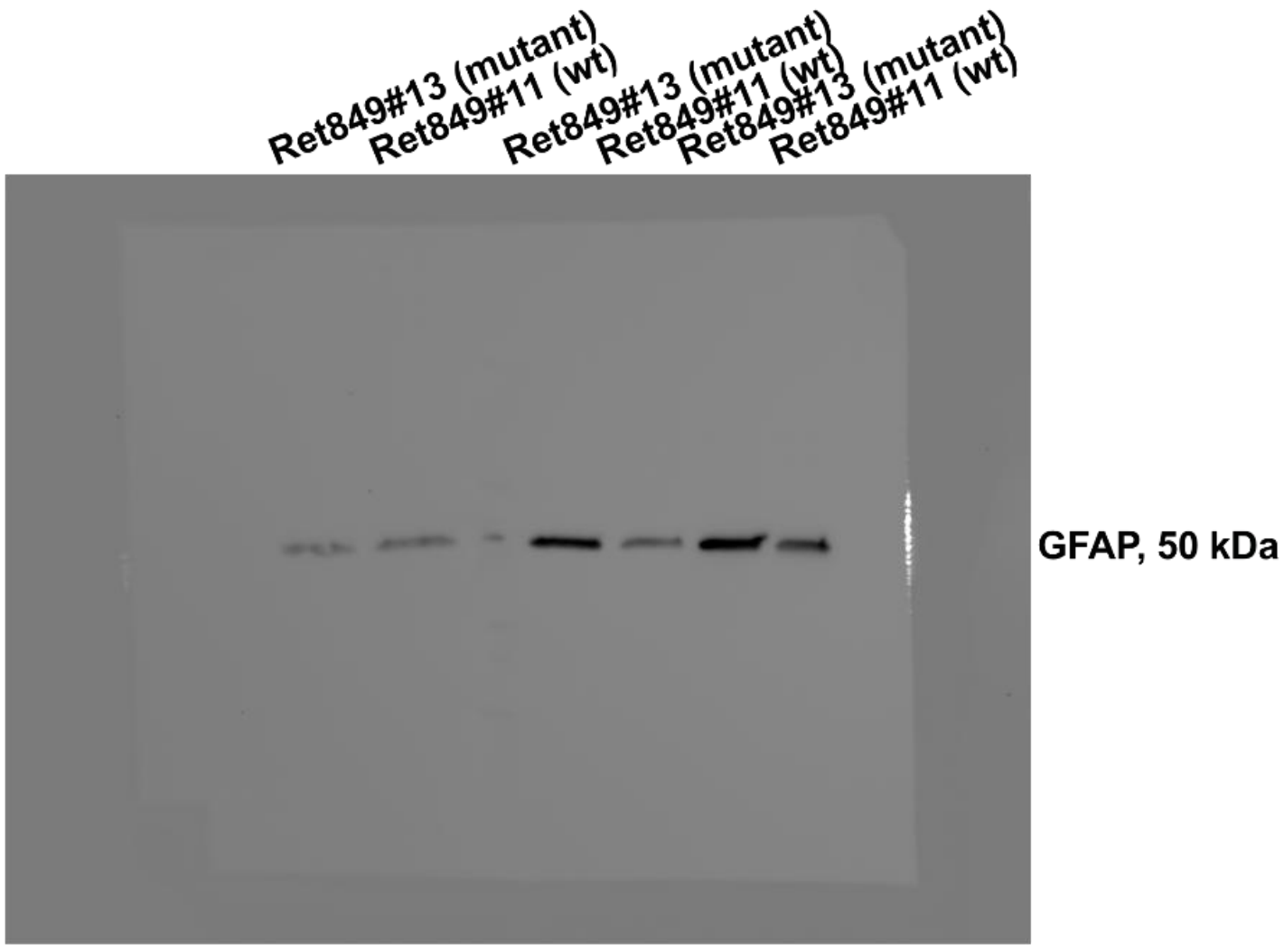

C)

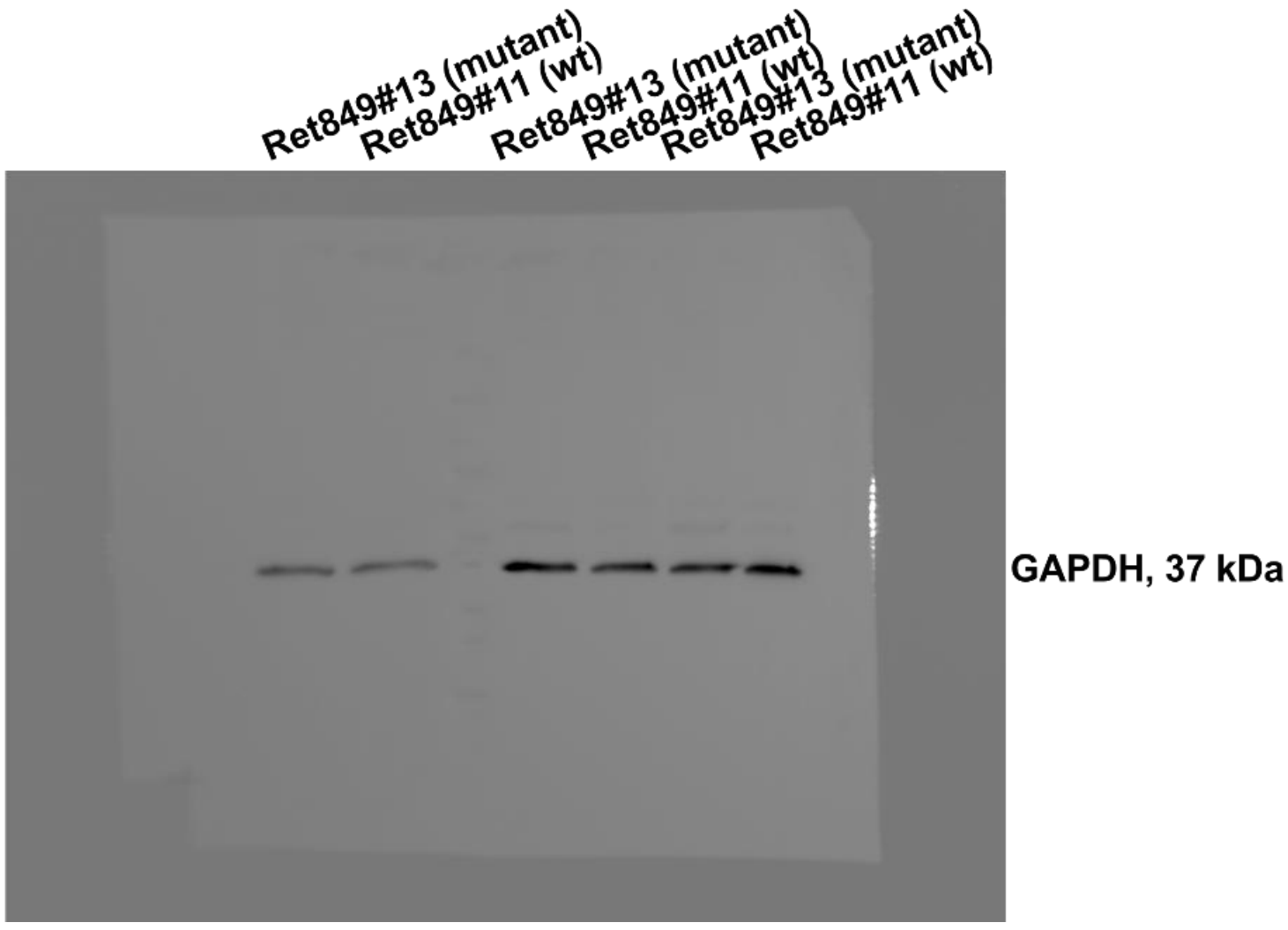

Supplemental Figure 19: Full sized original membrane images of western blot shown in Supplementary Figure 5. Membrane was probed for **A)** AQP4**; B)** GFAP; **C)** GAPDH.
